# *In vivo* CAR-T cell generation: a production-rate framework linking vector and cellular pharmacology

**DOI:** 10.3389/fimmu.2026.1939127

**Published:** 2026-09-18

**Authors:** Jing Hu, Dandan Zhu, Shanliang Zheng

**Affiliations:** 1 Zhengzhou Advanced Research Institute, Harbin Institute of Technology, Zhengzhou, China; 2 Henan Shenyou Medical Laboratory Co., Ltd, Zhengzhou, China; 3 School of Life Science and Technology, Harbin Institute of Technology, Harbin, China

**Keywords:** cancer immunotherapy, cellular pharmacology, gene delivery, *in vivo* CAR-T, lipid nanoparticle, production-rate framework, T-cell engineering, vector pharmacology

## Abstract

*In vivo* CAR-T cell generation is advancing rapidly within gene and cell therapy, but it is often framed as an “in-body” version of *ex vivo* CAR-T—a view that obscures fundamental pharmacological differences. We propose that *in vivo* CAR-T is better understood as a vector-to-cell pharmacological transition, in which vector pharmacology directly shapes cellular pharmacology: vector dose is only the administered input, whereas therapeutic cellular exposure is hypothesized to depend jointly on the rate, duration, and functional quality of CAR-T cells generated within the patient. Three generation-phase constraints — niche competition without lymphodepletion, founding-state lock-in, and antigen-encounter timing mismatch — define this transition and inform platform–indication matching. We develop this as a testable organizing hypothesis rather than a validated pharmacokinetic model, specifying which quantities are observed, which are inferred, and which are derived, together with four falsifiable predictions and the measurements required to evaluate them. “Production rate × functional window” is retained as a mnemonic for the two axes involved — the kinetics and duration of cellular generation, and the functional quality of the cells generated — rather than as an arithmetic product.

## Graphical Abstract

**Figure:**
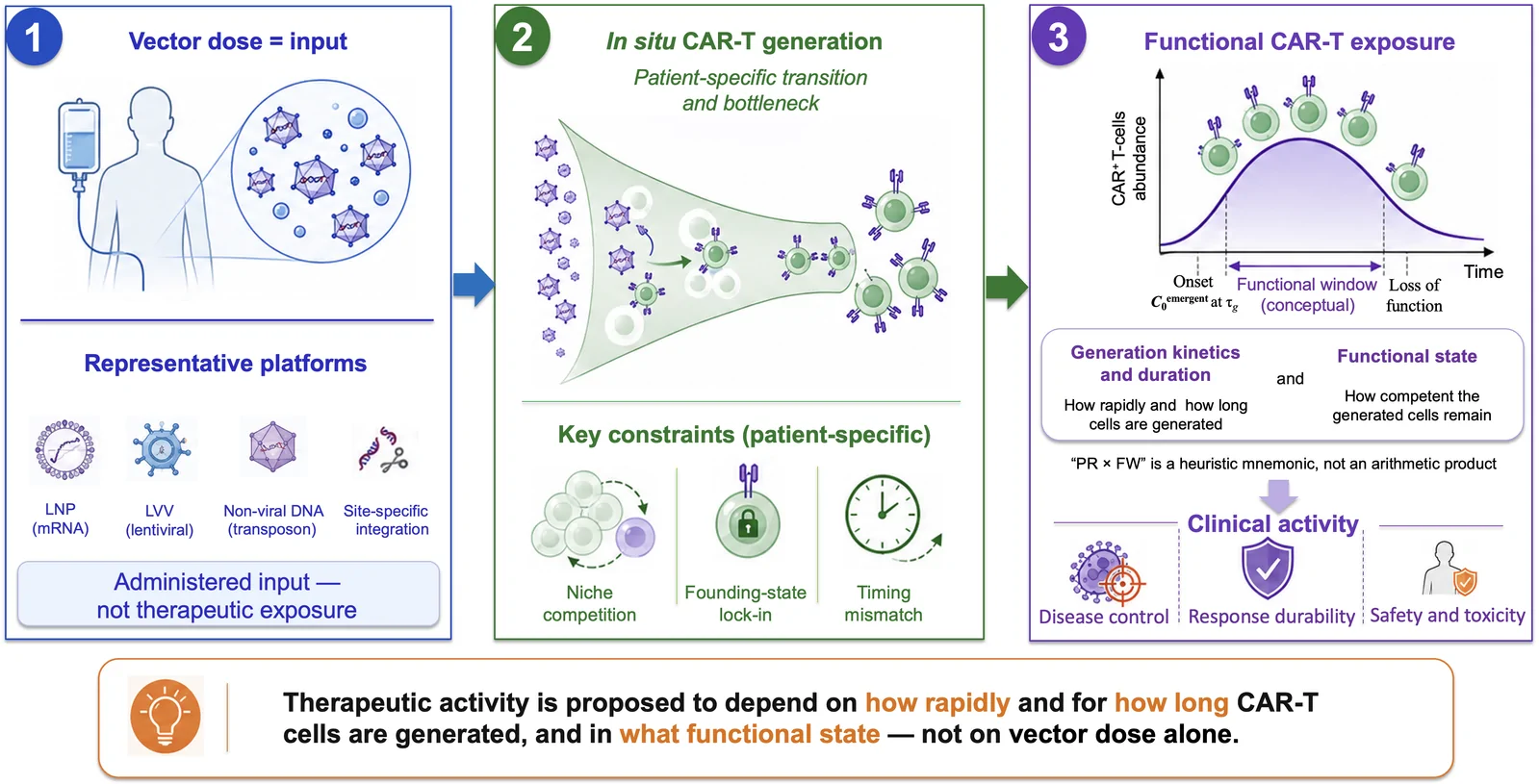

A production-rate framework for *in vivo* CAR-T cell generation. *In vivo* CAR-T therapy administers a vector dose as an input, whereas the pharmacologically active cellular population does not yet exist at the time of administration. Vector pharmacology shapes the patient- and platform-dependent conversion of that input into CAR-positive (CAR+) T cells through an *in situ* generation phase involving target-T-cell delivery, payload release and expression or integration, CAR surface display, early proliferation, survival, retention, trafficking, and host-dependent attrition. Three proposed generation-phase constraints — niche competition under lymphodepletion-free conditions, founding-state lock-in, and antigen-encounter timing mismatch — may limit the abundance and functional competence of the emerging CAR-T-cell population. The CAR+ T-cell abundance observed at the prespecified, platform-specific operational generation-phase boundary defines the emergent cellular boundary condition, *C*0emergent, for the canonical distribution, expansion, contraction, and persistence trajectory; generation and downstream expansion may overlap. Therapeutic activity is therefore hypothesized to depend jointly on the kinetics and duration of CAR-T-cell generation and on the functional state of the cells generated, rather than on nominal vector dose alone. “Production rate × functional window” is retained as a heuristic mnemonic for these jointly contributing axes rather than as an arithmetic product. The corresponding quantities are defined in **Box 1**, and the framework is advanced as a testable organizing hypothesis rather than a validated pharmacokinetic model.

Box 1 **Operationalizing the production-rate framework for *in vivo* CAR-T**

 To make the proposed framework operational rather than rhetorical, we distinguish directly observed cellular kinetics, latent biological determinants, and derived pharmacological quantities. The shorthand “production rate × functional window” (PR × FW) is retained only as a heuristic mnemonic for two axes along which *in vivo* CAR-T differs from cell-dose pharmacology: generation kinetics, including its duration, and functional quality. The multiplication sign does not imply dimensional equivalence or an actual arithmetic product. The framework is intended to specify what should be measured, what can reasonably be inferred, and what remains to be empirically validated; the corresponding reporting requirements are set out in **Table 1**.

A. Observed cellular kinetics, latent determinants, and derived pharmacological quantities

The framework specifies one observational primitive, $N_{\text{CAR},c}(t)$, from which a kinetic descriptor $r_{\text{app},c}(t)$ is derived; a set of latent biological determinants that are not independently estimated; and two derived pharmacological quantities, $C_{0}^{\text{emergent}}$ and $E_{\text{functional}}$.

(i) Observed cellular trajectory and apparent appearance rate.

The most direct cellular readout is the abundance of CAR-positive (CAR^+^) T cells in a specified sampled compartment: $N_{\text{CAR},c}(t)$, where *c* denotes the sampled compartment, such as peripheral blood or bone marrow. For clarity, the equations below are written in terms of absolute cell abundance [cells]; concentration-based implementations [cells·volume^-1^] are equally valid provided that units are carried consistently throughout. Direct enumeration of CAR^+^ T cells is preferred when available. Vector-copy measurements, including vector genomes per unit volume or vector-copy number per cell, should be regarded as supportive proxies rather than interchangeable measures of CAR^+^ T-cell abundance unless cell identity and residual free-vector contributions are resolved.

From the observed cellular trajectory, an apparent net appearance rate can be defined as

$$r_{\text{app},c}(t)=\frac{\text{d}N_{\text{CAR},c}(t)}{\text{d}t}$$

with units of cells·time^-1^ for an abundance-based implementation. Importantly, $r_{\text{app},c}(t)$ is an observational, compartment-dependent composite descriptor, not a direct measure of *de novo* CAR-T-cell production. It reflects the net consequence of new CAR acquisition, antigen-driven or homeostatic proliferation, cell death, loss of detectable CAR expression, and trafficking into and out of the sampled compartment. Being a net derivative, it may be negative during the contraction phase; this reflects its composite character and does not indicate negative generation. Its estimation depends on sampling density and should preferably rely on smoothed or model-based reconstruction of the cellular trajectory rather than numerical differentiation of sparse measurements.

(ii) Latent biological determinants.

Within this framework, the latent *de novo* CAR-acquisition rate is denoted $p_{c}(t)$, such that $r_{\text{app},c}(t)=p_{c}(t)+q_{c}(t)$, where $q_{c}(t)$ collects the net contribution of all non-generation processes— including proliferation, cell death, loss of detectable CAR expression, and trafficking into and out of compartment *c*—and may be positive or negative. $p_{c}(t)$ is the quantity that vector design, delivery efficiency, and target-cell tropism seek to raise, and is the production rate to which the framework’s name refers. It is not directly observable. No claim is made that $p_{c}(t)$ and $q_{c}(t)$ are separately identifiable from peripheral sampling alone; they are stated as latent biological determinants of the observed trajectory rather than as independently fitted parameters. This additive decomposition is conceptual rather than an estimable two-component model; without orthogonal data, only their sum is identified by the observed cellular trajectory.

Early survival and retention are not represented by a separate multiplicative *S*(*t*) term. This is primarily a structural and identifiability decision, rather than a dimensional one. Because *r*_app,_*_c_*(*t*) is defined as the net derivative of the observed cellular trajectory, the consequences of cell loss and compartmental retention are already embedded in that quantity. Multiplying $r_{\text{app},c}(t)$ by an additional survival term would therefore risk double-counting these processes and would introduce a component that is unlikely to be independently identifiable from the same peripheral-blood data.

Delivery attrition and payload constraints act primarily on *p_c_*(*t*); niche competition in the absence of lymphodepletion is represented principally through *q_c_*(*t*); and founding-state lock-in together with antigen-encounter timing mismatch are represented principally through *η*(*t*), defined below. These assignments identify the dominant pharmacological interpretation of each constraint rather than mutually exclusive biological pathways. This mapping is developed in Section 5.1.

Tissue sampling, bone-marrow measurements, molecular imaging, biodistribution studies, and mechanistic or compartmental models may progressively resolve these contributions as richer datasets become available.

(iii) Emergent generation-phase boundary condition.

We define $\tau_{g}$ as a prespecified, platform-specific analytical boundary for the period during which ongoing *de novo* CAR acquisition remains pharmacologically relevant. Generation and downstream expansion may overlap biologically; $\tau_{g}$ should therefore not be interpreted as a universal biological point at which generation abruptly stops and conventional cellular kinetics begin. Rather, it provides an operational reference for linking the generation phase to downstream cellular-kinetic analysis.

At this boundary, $C_{0}^{\text{emergent}}=N_{\text{CAR},c}(\tau_{g})$ represents the observed CAR^+^ cellular abundance at the operational generation-phase boundary and provides the initial cellular boundary condition for downstream modeling of distribution, expansion, contraction, and persistence.

The operational definition of $\tau_{g}$ should be supported by platform-appropriate vector, transgene, and cellular kinetic measurements and specified prospectively whenever possible. Criteria may differ between transient and integrating platforms and should therefore be justified rather than imposed universally. $C_{0}^{\text{emergent}}\;$is not interpreted as the cumulative number of cells generated *de novo*, because the observed abundance at $\tau_{g}$ also reflects proliferation, loss, and compartmental exchange during the generation phase.

Where a prespecified and independently justified scalar weighting rule is available at $\tau_{g}$, an exploratory weighted boundary quantity may additionally be reported:

$C_{0,\text{weighted}}=N_{\text{CAR},c}(\tau_{g})\cdot \eta (\tau_{g})$but this quantity is exploratory and should not replace the unweighted cellular measurement.

(iv) Functionally weighted cellular exposure.

The candidate derived exposure quantity proposed for future validation is based on the cellular state variable rather than on an appearance rate:

$$E_{\text{functional}}=\int_{0}^{T}N_{\text{CAR},c}(t)\;\eta (t)\;\text{d}t$$

with units of cells×time for an abundance-based implementation, or cells·volume^-1^ × time when concentration is used. Here *T* denotes a prospectively specified analysis horizon. Because $E_{\text{functional}}$ is truncation-dependent, *T* must be reported explicitly and held constant across compared groups or dose levels; where CAR^+^ T-cell persistence extends beyond *T*, this should be stated rather than extrapolated.

Computation of $E_{\text{functional}}$ or $E_{j}$ from discrete sampling requires prespecified reconstruction procedures appropriate to the properties and sampling schedules of $N_{\text{CAR},c}(t)$ and η(t), respectively. These may include linear or logarithmic trapezoidal integration for cellular abundance and prespecified interpolation or model-based reconstruction for functional measurements. The resulting product should then be integrated over the common analysis horizon. All reconstruction and integration rules should be declared *a priori*, because different choices may materially alter the estimated exposure.

This quantity is conceptually analogous to a concentration–time AUC when CAR^+^ T-cell concentration is the state variable, but it additionally incorporates time-dependent functional state. The dimensional distinction is important: integrating a rate over time yields a cell number, whereas integrating cellular abundance over time yields an exposure-like quantity. $E_{\text{functional}}$ is therefore advanced as a candidate pharmacological exposure metric, not as a validated clinical endpoint. Its value relative to vector dose, cellular *C*_max_, or conventional unweighted cellular AUC must be tested empirically.

B. η(t): a context-specific weighting construct, not a universal functional score η(t) represents a platform-, disease-, target-, and time-specific functional weighting construct. It is dimensionless and may be normalized to a prespecified scale, conventionally on [0, 1], so that $E_{\text{functional}}$ retains the units of an unweighted cellular exposure. Unless the scale has externally validated biological anchors, the resulting value should be interpreted as a context-specific weighted exposure rather than as a literal fraction of fully functional cells. Throughout, η(t) denotes functional state measured in the same compartment *c* as $N_{\text{CAR},c}(t)$; the compartment subscript is suppressed for readability. No universal CAR-T functional score is assumed. The same marker may carry different biological meaning depending on CAR design, disease context, antigen burden, treatment phase, and sampling compartment; high or low expression of an individual marker such as PD-1, TOX, TIM-3, LAG-3, or Ki-67 should therefore not be interpreted in isolation as equivalent to high or low CAR-T function.

At the present stage, functional state is better represented as a multi-domain characterization rather than forcibly collapsed into a single scalar:

**Table:** 

Domain	Representative readouts
CAR/signaling competence	CAR surface density; durability of expression; signaling responsiveness
Differentiation state	stem-like or memory fractions; terminal differentiation
Effector competence	cytotoxicity; cytokine polyfunctionality; target-cell killing
Proliferative and cellular fitness	proliferative reserve; metabolic fitness; exhaustion-associated phenotype

Spatial localization and tissue accessibility should be reported separately as compartmental pharmacokinetic or pharmacodynamic variables rather than incorporated into η(t).

When a scalar representation is required, η(t) may be normalized to a prespecified dimensionless scale within a defined platform and disease context. The domains included, their weighting, and the normalization rule must be declared *a priori* or validated independently; they should not be optimized retrospectively against clinical response in the same cohort.

Until such composite weighting schemes are prospectively validated, domain-specific exposures may be preferable: $E_{j}=\int_{0}^{T}N_{\text{CAR},c}(t)\;\eta_{j}(t)\;\text{d}t$, where *j* denotes an individual functional domain. Evidence from *ex vivo* CAR-T studies that infusion-product immunophenotype and T-cell fitness contribute prognostic information alongside *in vivo* cellular expansion supports functional characterization in principle, but does not establish a universal weighting rule for *in vivo* CAR-T (78).

C. Falsifiable predictions.

The production-rate framework is intended as a testable organizing hypothesis. It therefore generates predictions that can support, refine, narrow, or falsify it.

Prediction 1 — Vector dose is a weak predictor of emergent cellular output and downstream exposure.

Across dose levels, models based on nominal vector dose alone will show poorer out-of-sample prediction of $C_{0}^{\text{emergent}}$ and downstream cellular exposure — assessed, where sample size permits, by prespecified internal validation, cross-validation, or an independent cohort rather than by apparent in-sample fit — than models that additionally incorporate prespecified generation-phase, host, and disease covariates. Within a nominal dose cohort, observed interpatient variability in these cellular outcomes would provide supporting evidence that nominal dose is insufficient as an individual-level predictor, although such within-cohort variability would not by itself estimate a dose effect. If dose-only models perform comparably under out-of-sample evaluation, the premise that a patient-specific generation phase materially separates administered vector dose from cellular exposure would be weakened.

Prediction 2 — Functional characterization adds predictive value beyond cell number alone.

When functional domains and weighting rules are prespecified or independently validated, functionally weighted cellular exposure should improve out-of-sample prediction of clinical activity relative to cellular $C_{max}$ or unweighted cellular exposure alone. In the near term, this prediction is most directly testable in its domain-specific form using $E_{j}$, which requires no composite weighting rule and therefore avoids circularity between construction and validation. If functional weighting repeatedly fails to add predictive value across domains, the need for an explicit η(t) construct should be reconsidered.

Prediction 3 — Platform- and product-specific kinetic signatures.

Within a product family evaluated in comparable disease and treatment contexts, joint signatures of CAR^+^ T-cell appearance kinetics, the vector and transgene kinetics used to support the operational definition of *τ_g_*, cellular persistence, and functional-state trajectories should be reproducible across cohorts. *τ_g_* itself is an analytical convention rather than an observed property and is therefore not a component of such signatures. Reproducibility within a product family would support this prediction, whereas reproducibility only at the individual-product level would narrow rather than invalidate it. Generalization to broader platform classes would require independent demonstration. If prespecified joint signatures fail to reproduce out of sample within the same product family, or if platform or product identity provides no incremental discriminatory information beyond host, disease, and treatment-context variables, this prediction would be falsified. Demonstration of reproducible signatures could provide a quantitative basis for platform–indication matching and help inform — but should not automatically determine — regulatory differentiation.

Prediction 4 — Therapeutic activity and toxicity occupy related but non-identical kinetic windows.

The exposure metrics and time horizons that best predict therapeutic activity are hypothesized to differ systematically from those that best predict acute toxicity, as has been observed in *ex vivo* CAR-T (78). Functionally weighted cellular exposure over a prospectively specified efficacy-relevant horizon is proposed as a candidate predictor of therapeutic activity, whereas early vector exposure, the rate of CAR^+^ T-cell expansion, inflammatory biomarkers, or their combinations may better predict acute toxicity. The metric and time horizon selected for efficacy should therefore not be assumed to be optimal for toxicity, or vice versa. If the same metric and time horizon consistently predict both outcomes and early kinetic or inflammatory variables provide no incremental predictive value, this prediction would be falsified.

If these predictions are repeatedly falsified, the production-rate framework should be revised, narrowed, or replaced. If they are supported, the results would justify viewing *in vivo* CAR-T as a distinct vector-, host-, and disease-dependent pharmacological context in which dose selection and pharmacodynamic reporting cannot rely on vector dose alone.

## 1 Introduction: the vector-to-cell pharmacological transition in *in vivo* CAR-T

Gene and cell therapy has entered a phase in which the boundary between vector and cell product is becoming increasingly fluid. Chimeric antigen receptor (CAR) T-cell therapy represents the most clinically mature example of this convergence. Autologous *ex vivo* CAR-T products have reshaped the treatment of B-cell malignancies and multiple myeloma, with multiple products approved in the United States (1). Yet broad access remains constrained by the bespoke nature of these therapies: leukapheresis, centralized manufacturing, cold-chain logistics, high list prices, and vein-to-vein intervals of several weeks, during which some eligible patients progress or die before infusion (2–4). These challenges are often framed as problems of manufacturing and access, but they also expose a deeper pharmacological limitation of manufactured cellular products: cellular exposure cannot be fully prescribed at the point of administration.

Even when an *ex vivo* CAR-T product reaches the patient on schedule, it enters as a fixed, one-time cellular bolus. Its subsequent distribution, expansion, contraction, and persistence are shaped by host- and disease-specific conditions that cannot be fully controlled during manufacturing (5, 6). This disconnect between manufactured input and *in vivo* behavior is especially evident in solid tumors, where stromal exclusion, abnormal vasculature, chronic antigen exposure, and immunosuppressive cues can limit infiltration, survival, and function of infused CAR-T cells (5, 7, 8). Point-of-care manufacturing, shortened culture, and allogeneic platforms may reduce cost and logistical delay, but they do not by themselves resolve this pharmacological disconnect between administered cell dose and functional cellular exposure.

The field has therefore moved toward a fundamentally different architecture: *in vivo* CAR-T cell generation. In this approach, engineered vectors deliver CAR-encoding genetic material directly to T cells inside the patient, converting the body from a recipient of a manufactured cell product into the site of cell-product generation. A translational arc has emerged from murine proof-of-concept showing that transient, non-genomic CAR expression can be therapeutically meaningful (9), to non-human primate validation of *in situ* B-cell depletion (10), to first-in-human reports in relapsed/refractory multiple myeloma using targeted lentiviral platforms such as ESO-T01 and KLN-1010 (11–13) and, on a non-integrating platform, a CD8-targeted mRNA-LNP in CD19^+^ B-cell lymphoma reported in abstract form (84), and, most recently, to site-specific *in vivo* transgene integration into the *TRAC* locus in solid-tumor models (14). These studies should be interpreted as early proof of clinical feasibility rather than definitive evidence of efficacy, given their small sample sizes, single-arm designs, and limited follow-up. Nevertheless, their breadth across fibrosis, autoimmunity, hematologic malignancy, and solid-tumor models suggests that *in vivo* CAR-T is not merely a manufacturing alternative, but a cross-disease platform for programmable immune-cell generation (15–17).

This rapid technological progress has outpaced the conceptual framework used to interpret it. *In vivo* CAR-T is often described as an “in-body” version of *ex vivo* CAR-T (18, 19). That framing is useful for explaining access and manufacturing advantages, but it understates the central pharmacological shift. In *ex vivo* CAR-T, vector engineering occurs upstream of clinical administration, and the patient receives a characterized cellular product. In *in vivo* CAR-T, by contrast, the administered product is the vector, and the therapeutic cell population is generated inside the patient through a host-dependent conversion process. Vector pharmacology therefore shapes cellular pharmacology: biodistribution, target-cell uptake, payload expression, integration status, immunogenicity, persistence, and redosability collectively determine the rate, durability, safety profile, and functional quality of the CAR-T cells that emerge (**Figure 1**).

**Figure 1:**
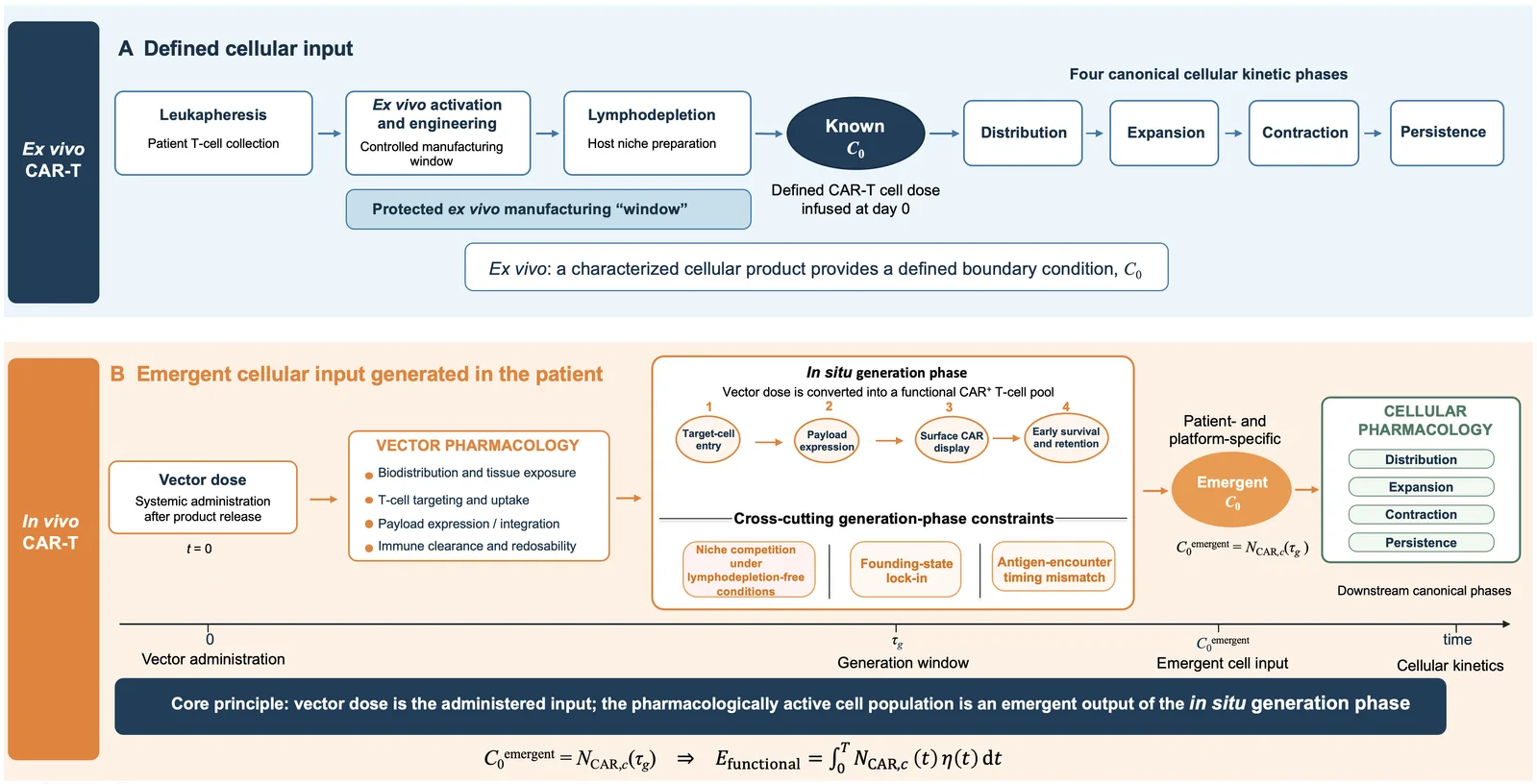
From vector pharmacology to cellular pharmacology in *in vivo* CAR-T generation. **(A)** In *ex vivo* CAR-T therapy, leukapheresis, controlled activation and genetic engineering, and lymphodepletion precede infusion of a characterized cellular product. The infused CAR-T-cell dose provides a defined cellular boundary condition, *C*_0_, from which the canonical distribution, expansion, contraction, and persistence phases proceed. **(B)** In *in vivo* CAR-T therapy, the administered product is a vector. Vector biodistribution, T-cell targeting and uptake, payload expression or integration, and immune clearance determine the upstream *in situ* generation phase. Platform-specific redosability determines whether this process can be reinitiated by repeat dosing. Through this generation process, a functional CAR^+^ T-cell pool emerges within the patient. This phase is shaped by three proposed, cross-cutting constraints: niche competition under lymphodepletion-free conditions, founding-state lock-in by the host environment, and antigen-encounter timing mismatch. In the framework developed in Section 5.1 and **Box 1**, delivery and payload processes are represented principally through the latent *de novo* CAR-acquisition rate, *p_c_* (*t*); niche competition is represented principally through the net non-generation term, *q_c_* (*t*); and founding-state lock-in and antigen-encounter timing mismatch are represented principally through the functional-state weighting construct, *η*(*t*). These assignments indicate dominant interpretations rather than mutually exclusive pathways. The CAR^+^ T-cell abundance observed at the prespecified operational boundary of the generation phase, *τ_g_*, defines the emergent cellular boundary condition, *C*_0_^emergent^ = *N*_CAR,_*_c_*(*τ_g_*), which provides the boundary condition for the downstream distribution, expansion, contraction, and persistence trajectory. The candidate functionally weighted cellular exposure accumulated over the prespecified analysis horizon is defined in **Box 1**. These phases are separated conceptually for analytical purposes but may overlap biologically.

We propose that this transition is best understood as a shift from cell-dose pharmacology to production-rate pharmacology. Vector dose is only the administered input; cellular exposure is hypothesized to depend jointly on the rate, duration, and functional quality of CAR-T cells generated within the patient. Throughout, *production rate* denotes the latent rate at which T cells within the patient newly acquire detectable CAR expression meeting a prespecified assay criterion. It is not directly observable and is distinct from the apparent rate of CAR^+^ T-cell appearance returned by peripheral sampling, which is a net composite of generation, proliferation, loss, and trafficking; the two are defined and separated in **Box 1**. This reframing has three linked dimensions. First, *in vivo* CAR-T requires a kinetic framework in which the cellular boundary condition *C*_0_^emergent^ (**Box 1**) emerges from an upstream, patient-specific *in situ* generation phase rather than from an infused cell dose. Second, it imposes a platform-specific safety geometry — the characteristic distribution of risk across time and mechanism that follows from a given platform’s persistence, reversibility, and redosability — because vectors range from transient, redosable RNA systems to durable integrating platforms that may require active fail-safe design (6, 18, 19). Third, it creates a vector-dependent regulatory interface between gene-therapy and cell-therapy pathways, with implications for chemistry and manufacturing, insertional risk, redosing strategy, and long-term follow-up (20–22).

This framework is anchored in three generation-phase constraints. First, *in vivo*-generated CAR-T cells must expand without the cytokine-niche opening normally created by lymphodepletion — that is, lymphodepleting conditioning transiently clears endogenous lymphocytes that compete for the homeostatic γc cytokines IL-7 and IL-15, increasing their availability to newly expanding T cells (23, 24). Second, they acquire their founding functional state within the host inflammatory milieu rather than during a protected *ex vivo* manufacturing window, potentially biasing early chromatin remodeling toward dysfunctional trajectories — a constraint we term *founding-state lock-in*, since the differentiation program established during the first days of CAR expression becomes progressively resistant to reversal (25–29). Third, nascent CAR-expressing T cells may encounter high antigen burden before CAR surface density and downstream signaling competence are fully established, and this antigen-driven signaling is superimposed on *tonic signaling* — antigen-independent signaling arising from spontaneous clustering or aggregation of the CAR itself — which begins as soon as the receptor reaches the cell surface (30–32). Each concept is grounded in established T-cell biology but remains to be systematically tested in the *in vivo* setting. Together, these constraints support a testable organizing hypothesis: that prespecified generation-phase variables will improve out-of-sample prediction of cellular exposure and clinical activity relative to nominal vector dose alone. **Box 1** states this hypothesis as four falsifiable predictions and defines the quantities involved, and **Table 1** specifies the measurements required to evaluate them. “Production rate × functional window” is used throughout as a mnemonic for the two axes concerned — the kinetics and duration of cellular generation, and the functional quality of the cells generated — and not as an arithmetic product. The remainder of this Review develops this framework, beginning with how vector pharmacology determines cellular pharmacology before any downstream therapeutic advantage can be realized.

**Table 1:** Reporting checklist for early-phase *in vivo* CAR-T studies.

Priority	Item	Rationale
Core	Vector dose, together with platform-appropriate vector kinetics, biodistribution, and, where relevant, shedding	Anchors the administered input, supports a platform-specific operational definition of $\tau_{g}$, and provides the exposure and containment data required for regulatory characterization
	Longitudinal CAR^+^ T-cell abundance or concentration, with sampled compartment explicitly specified	Provides $N_{\text{CAR},c}(t)$, the observational primitive from which all derived quantities are computed
	Platform-adapted early sampling density sufficient to reconstruct the appearance, expansion, and peak phases, with the sampling window justified according to the expected vector and transgene kinetics (for example, over the first 3–4 weeks for selected transient platforms)	Sparse sampling makes $r_{\text{app},c}(t)$ and $C_{0}^{\text{emergent}}$ unreliable
	Prospectively justified operational definition of $\tau_{g}$ and the corresponding $C_{0}^{\text{emergent}}$	Prevents *post hoc* selection of the generation-window boundary
	CAR expression per cell, where technically feasible	Distinguishes cell number from per-cell CAR/signaling competence
	Baseline T-cell composition and absolute lymphocyte count	Candidate host determinant of target-cell availability and achievable $p_{c}(t)$
	Antigen or disease burden at vector administration	Determines antigen-encounter timing relative to CAR acquisition
	Safety and inflammatory readouts, including serum cytokines	Enables evaluation of temporal relationships between vector exposure, CAR^+^ T-cell kinetics, systemic inflammation, and treatment-emergent toxicity
	Prospectively specified dose-escalation, de-escalation, cohort-expansion, and schedule-modification rules, including any safety and pharmacodynamic decision criteria	Enables accumulating cellular and pharmacodynamic data to inform dose selection without *post hoc* adaptation; unvalidated latent or derived quantities should not serve as standalone dose-assignment variables
	Anti-vector immunity, where biologically relevant	Constrains $p_{c}(t)$ and redosability
	Conventional kinetic measures, including cellular *C*_max_ and unweighted cellular exposure	Required comparators for Predictions 1 and 2
Exploratory	Bone-marrow or tissue-specific CAR-T kinetics	Helps distinguish compartmental exchange from other contributions to $q_{c}(t)$
	Molecular imaging or other spatial biodistribution approaches	Addresses trafficking separately from η(t)
	Integration-site analysis and clonal tracking, for integrating platforms	Longitudinal clone tracking helps distinguish continued *de novo* CAR acquisition from expansion of previously generated clones
	Anti-CAR immune responses	Additional determinant of $q_{c}(t)$
	Multi-domain functional phenotyping at prespecified early/near-peak and persistence time points	Supplies domain-specific functional measurements $\eta_{j}(t_{k})$ at prespecified sampling times and supports estimation of $E_{j}\;$when the longitudinal reconstruction rule is prespecified
	Paired baseline and early post-acquisition single-cell transcriptional and, where feasible, epigenetic or metabolic profiling	Tests whether baseline target-cell states and early post-acquisition molecular trajectories are consistent with the founding-state lock-in hypothesis

The immediate goal of the framework is not to require estimation of every latent biological process, but to improve the information content of early clinical datasets.

Together, longitudinal vector and transgene kinetics, CAR^+^ T-cell abundance, cellular persistence, and functional-state measurements provide the joint data required to test the within-product reproducibility and cross-platform generalizability proposed in Prediction 3. Where sufficient data are available, $E_{\text{functional}}$ or domain-specific $E_{j}$ values should be reported alongside—not in place of—vector dose, cellular $C_{max}$, and unweighted cellular exposure, with the analysis horizon *T* stated. Their incremental out-of-sample predictive value for therapeutic activity and toxicity should then be tested rather than assumed.

## 2 The double barrage: disease-context barriers to *in vivo* CAR-T generation

The vector-to-cell pharmacological transition described in the Introduction must occur within a biological environment that the vector cannot fully shape. *In vivo* CAR-T translation therefore faces what we term the double barrage: two sequential layers of attrition that compress into a single developmental window during which vector pharmacology is being converted into cellular pharmacology. The first—pharmacokinetic attrition during vector delivery (Section 2.1) — is unique to *in vivo* generation and reflects the vector’s biodistribution and target-cell tropism. The second—the suppressive realities of the tumor microenvironment (TME) (Section 2.2) — is shared with *ex vivo* CAR-T but is encountered under fundamentally different conditions: rather than confronting the TME with mature effector cells expanded under controlled *ex vivo* conditions, *in vivo*-generated CAR-T cells must navigate it during the window when their functional phenotype is still being established.

This double-barrage framing predicts a disease-context asymmetry that should influence platform selection. In hematologic malignancies, *in vivo* CAR-T applications may be dominated by Layer 1, because systemic vectors, nascent CAR^+^ T cells, and target cells converge in accessible compartments such as blood, bone marrow, and secondary lymphoid organs. These environments are not immunologically neutral, but they are more accessible to systemically administered vectors and less structurally restrictive than solid tumors. Solid-tumor applications, by contrast, must cross both Layer 1 and Layer 2 simultaneously: vectors face delivery attrition, while the cells they generate must establish functional competence under stromal, hypoxic, and immunosuppressive pressures from the moment of birth. This asymmetry may help explain why first-in-human experience — including ESO-T01 and KLN-1010 in BCMA-expressing multiple myeloma and a CD8-targeted mRNA-LNP in CD19^+^ B-cell lymphoma — has so far accumulated in hematologic indications, whereas solid-tumor *in vivo* CAR-T applications remain largely preclinical (11–14, 84).

Beyond these two established layers of barrier, we propose three previously unappreciated kinetic constraints that define the *in situ* generation phase itself: niche competition under lymphodepletion-free conditions (Section 2.3), founding-state lock-in by the *in vivo* microenvironment (Section 2.4), and antigen-encounter timing mismatch (Section 2.5). Together these constraints specify why *in vivo* CAR-T generation is not simply a more accessible form of *ex vivo* manufacturing, but a distinct pharmacological process in which vector attributes directly shape cellular fate during a developmentally critical window (**Figure 2**).

**Figure 2:**
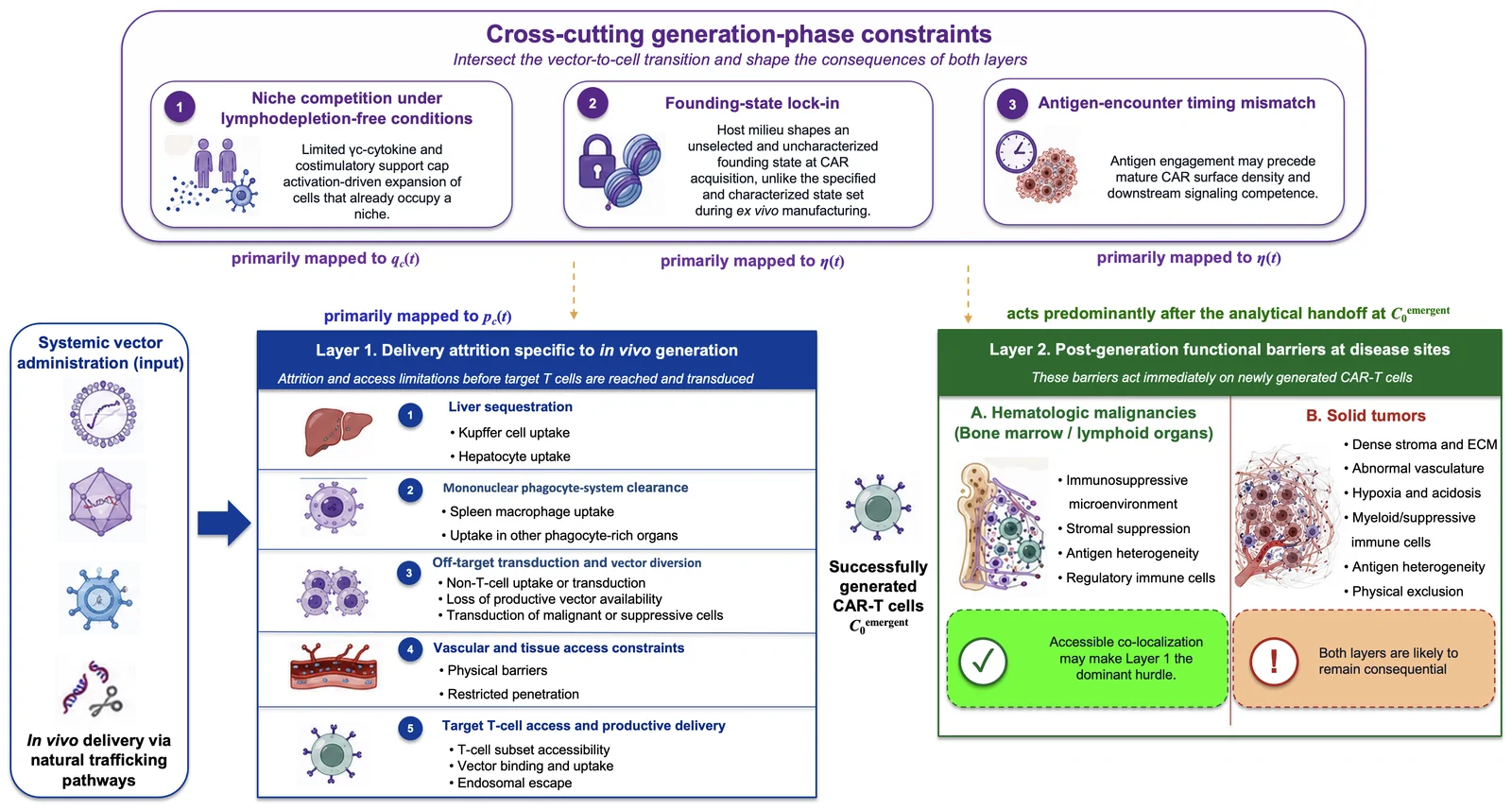
The double barrage and disease-context asymmetry of *in vivo* CAR-T generation. Layer 1. Systemically administered vectors encounter delivery attrition before productive T-cell engineering, including hepatic and mononuclear phagocyte-system sequestration, off-target uptake or transduction, restricted vascular and tissue access, and inefficient entry into target T cells. Layer 2. CAR-T cells that are successfully generated subsequently encounter disease-site barriers, including stromal restriction, abnormal vasculature, hypoxia, suppressive immune populations, and antigen heterogeneity. The relative contributions of these layers are expected to differ across disease contexts. **(A)** In hematologic malignancies, vector biodistribution, target T cells, and antigen-bearing cells may co-localize in relatively accessible blood, bone-marrow, and lymphoid compartments; delivery attrition may therefore represent the dominant hurdle. **(B)** In solid tumors, both delivery attrition and post-generation functional barriers are likely to remain consequential. Three proposed generation-phase constraints intersect the vector-to-cell transition and influence how delivery attrition is converted into the number and functional state of CAR-T cells entering disease-site compartments. Under lymphodepletion-free conditions, and because the transduced cells are endogenous and already occupy a homeostatic niche, limited γc-cytokine availability and costimulatory support may impose a ceiling on activation-driven CAR-T-cell expansion rather than physically excluding newly generated cells from the niche. Founding-state lock-in denotes the possibility that the host milieu shapes an unselected and uncharacterized functional state at the time of CAR acquisition, in contrast to ex vivo manufacturing, in which the founding state is a specified and characterized variable. Antigen-encounter timing mismatch arises when nascent CAR-T cells engage antigen before CAR surface density and downstream signaling competence are fully established. Within the framework developed in Section 5.1 and **Box 1**, delivery attrition is mapped principally to the achievable magnitude and temporal profile of the latent *de novo* CAR-acquisition rate *p_c_* (*t*); niche competition is mapped principally to the net non-generation term *q_c_*(*t*); and founding-state lock-in and antigen-encounter timing mismatch are mapped principally to the functional-state weighting construct *η*(*t*). *C*_0_^emergent^ marks an analytical handoff rather than a strict biological separation. Because generation and downstream cellular responses may overlap, disease-site barriers may also influence *q_c_*(*t*) and *η*(*t*) during the operational generation window.

### 2.1 Layer 1: delivery attrition specific to *in vivo* generation

The first layer comprises delivery attrition—a hurdle entirely absent from the *ex vivo* pipeline, where vector–cell encounter is engineered under controlled laboratory conditions. Systemically administered vectors face substantial pharmacokinetic losses before encountering a target T cell, and these losses are themselves vector-determined (18, 33). The liver acts as a dominant biodistribution sink: systemically infused LNPs rapidly bind serum apolipoprotein E, which targets them to hepatocyte LDL receptors, while Kupffer cells intercept additional particles before they reach extrahepatic tissues (34, 35). Engineering targeting selectivity adds a second constraint. T-cell-restricted ligands (anti-CD3, anti-CD5, or anti-CD8 antibodies and nanobodies) are required to minimize off-target transduction (9, 10, 36, 37); absent this specificity, gene transfer into bystander cells—including tumor-associated macrophages, or the malignant cells themselves—can produce paradoxical outcomes ranging from amplified immunosuppression to CAR-mediated epitope masking on tumor cells, accelerating antigen escape (38, 39).

Off-target uptake should be distinguished from off-target transgene expression. Uptake by hepatic or phagocytic cells primarily reduces the productive vector available for transduction and therefore acts on the generation rate itself, whereas transduction of malignant cells may produce CAR-mediated epitope masking and antigen escape (36). Expression in myeloid or antigen-presenting cells, or in non-T lymphocyte populations, could instead create ectopic signaling, alter immune activation, or generate unintended CAR-expressing effector populations; the biological significance of these outcomes remains platform- and construct-dependent and, to our knowledge, has not been systematically compared.

A related consideration concerns the targeting ligand itself. CD3-directed ligands may alter target-cell activation or phenotype, as demonstrated with anti-CD3-decorated LVV and EDV platforms (14, 36, 37). The magnitude and quality of this effect are likely to depend on ligand format and agonistic activity. Because the resulting activation state may shape the founding state of cells that acquire a CAR (Section 2.4), this property should be characterized separately from delivery efficiency rather than inferred from transduction yield.

### 2.2 Layer 2: functional barriers shared with the *ex vivo* paradigm

Even when delivery attrition is overcome and CAR^+^ T cells are generated *in situ*, they immediately confront the second layer: the classical functional barriers of the solid TME (5, 7). Dense extracellular matrix, aberrant vasculature, and regional hypoxia restrict T-cell infiltration and survival (40–42); intratumoral regulatory T cells, tumor-associated macrophages, and myeloid-derived suppressor cells elaborate inhibitory cytokines that blunt effector function (43, 44); and pronounced antigen heterogeneity drives antigen escape (38, 45). *Ex vivo* CAR-T programs increasingly address these barriers through armored CAR designs co-expressing pro-inflammatory cytokines or dominant-negative TGF-β receptors (46, 47). Translating armoring to the *in vivo* setting, however, is constrained by vector-specific payload economics: AAVs face a strict packaging limit of ~4.7 kb (48); LNPs can co-encapsulate multiple mRNAs but introduce stoichiometric control challenges across cargos and dose-dependent cytotoxicity scaling with total RNA load (34); lentiviral vectors have a practical ~8–10 kb ceiling beyond which titer drops sharply. These platform-specific design constraints — not a generic “packaging problem” — narrow the design space for armored *in vivo* CAR-T relative to *ex vivo* counterparts (19), a constraint we develop further in Section 4.

### 2.3 Generation-phase constraint 1: niche competition under lymphodepletion-free conditions

The first kinetic constraint is niche competition under lymphodepletion-free conditions. Effective expansion of *ex vivo* CAR-T cells depends on lymphodepleting preconditioning, which clears endogenous lymphocytes that otherwise act as cellular sinks for the homeostatic γc cytokines IL-7 and IL-15 (23, 24, 49). Removal of these endogenous γc-cytokine-responsive cells, by either antibody depletion or genetic ablation, augments adoptively transferred CD8^+^ T-cell function to a degree comparable to chemotherapy-mediated lymphoablation (24). Lymphodepletion thus opens the cytokine niche and reduces competition for antigen-presenting cell (APC) contacts.

A major translational appeal of many *in vivo* CAR-T strategies is the potential to reduce or omit lymphodepletion — and with it, the conditioning effects that support *ex vivo* CAR-T expansion. However, the form of niche competition faced by *in vivo*-generated CAR-T cells differs in an important respect from that faced by adoptively transferred *ex vivo* CAR-T cells. *Ex vivo* CAR-T cells are exogenous, entering the patient as a population that does not yet occupy any homeostatic niche and must establish access to IL-7, IL-15, and APC contacts from the moment of infusion. *In vivo*-generated CAR-T cells, by contrast, are endogenous T cells that already occupy a homeostatic niche prior to transduction; their pre-CAR baseline already includes some access to γc cytokines and APC interactions. The relevant question is therefore not whether transduced cells can secure niche access *de novo*, but whether their pre-existing niche access is sufficient to support the CAR-driven proliferative burst once antigen engagement occurs.

This refined framing yields a specific prediction. Newly transduced CAR^+^ T cells, upon antigen encounter, undergo activation-driven proliferation that places acute additional demand on γc cytokine availability — demand that may exceed pre-CAR baseline access. Without lymphodepletion, this additional demand competes with the maintenance demands of the pre-existing circulating and tissue-resident T-cell compartments. The constraint is therefore not absolute exclusion from the niche but a ceiling on activation-driven niche expansion — a kinetic limit that cellular pharmacokinetic models derived from lymphodepleted *ex vivo* cohorts do not capture.

γc cytokine availability is only one component of this niche. The two paradigms also differ in where the activation and costimulatory signals that sustain proliferation originate. In *ex vivo* manufacturing these signals are supplied synthetically: anti-CD3/CD28 beads or artificial antigen-presenting constructs deliver receptor engagement and CD28 costimulation at defined stoichiometry and duration, and γc cytokine support is added at controlled concentration, so that the endogenous APC compartment is bypassed entirely. Whatever the state of the patient’s dendritic cells and myeloid compartment, the manufacturing environment substitutes for them. *In vivo*-generated CAR-T cells have no such substitute: the costimulatory and cytokine signals available to support expansion and persistence after CAR acquisition are whatever the host lymphoid environment provides, and are influenced by dendritic-cell frequency and maturation state as well as by myeloid composition. In the terms of Section 5.1, this contribution acts principally on the net non-generation term *q_c_*(*t*) alongside the γc cytokine constraint described above.

Whether the endogenous APC compartment is principally supportive or principally limiting in this setting is an empirical question rather than one that can be settled by argument, and it is likely to be patient-dependent. Several considerations suggest that the limiting case deserves attention. Patients eligible for *in vivo* CAR-T are typically heavily pretreated, and prior cytotoxic therapy can deplete and functionally impair dendritic-cell compartments. Tumor-derived factors drive dendritic-cell dysfunction and skew myeloid differentiation toward suppressive phenotypes, reducing costimulatory availability while increasing inhibitory signaling (50, 51). If lymphodepleting conditioning is omitted — one of the principal practical attractions of the *in vivo* approach — the cytokine surge that ordinarily supports early expansion is also absent, so that costimulatory and cytokine support may be diminished simultaneously. An important qualification limits how far this dependence extends: second-generation CAR constructs incorporate a CD28 or 4-1BB endodomain that delivers costimulation cell-intrinsically upon antigen engagement and does not require APC-derived ligands (52). Once a CAR is expressed and encounters antigen, the construct itself supplies this signal. The dependence described here is therefore concentrated in the period around CAR acquisition and in the maintenance of the broader cytokine environment, rather than in CAR-mediated effector activation itself — which is to say, precisely in the generation window that *ex vivo* manufacturing removes from the patient and that *in vivo* generation returns to it. Baseline dendritic-cell frequency and maturation state and myeloid composition are measurable, and are candidate host covariates of the kind specified in Prediction 1 (**Box 1**).

Early clinical signals of ESO-T01 and KLN-1010 in relapsed/refractory multiple myeloma indicate that this niche competition is not absolute (11–13). Heavily pretreated multiple myeloma patients are typically immunosuppressed and lymphopenic from prior lines of therapy, conditions that may transiently relieve cytokine sink pressure. This clinical context aligns with the refined framing: in heavily pretreated patients, the competing endogenous cytokine sink may be reduced, making the additional activation-driven demand of CAR engagement easier to satisfy. Whether this reflects an indication-specific advantage that may not generalize to immunocompetent patients (e.g., autoimmune indications) or to solid tumors — where endogenous T-cell counts are typically intact — remains an open question.

### 2.4 Generation-phase constraint 2: founding-state lock-in by the *in vivo* microenvironment

T-cell exhaustion is now understood not as a late functional decline but as a chromatin-level developmental program established within the first days of antigen encounter (25). Philip et al. demonstrated that tumor-specific CD8^+^ T cells differentiate through two discrete chromatin states: an early plastic dysfunctional state from which cells can be rescued, and a later fixed dysfunctional state resistant to reprogramming, including by checkpoint blockade (26). Massive chromatin remodeling occurs by day 5 after tumor antigen encounter, with a second wave between days 7 and 14, after which little further remodeling occurs (26).

TOX is a central regulator of this transition. Converging 2019 studies established TOX as a key transcriptional and epigenetic driver of CD8^+^ T-cell exhaustion (27–29). However, TOX-dependent exhaustion should not be viewed as purely pathological. Scott et al. showed that TOX-deleted tumor-specific T cells, despite a non-exhausted immunophenotype, failed to persist in tumors, leading them to propose that TOX-induced exhaustion functions as a survival program that limits overstimulation and activation-induced cell death under chronic antigen exposure (29). This dual role is directly relevant to *in vivo* CAR-T: early CAR expression in an inflammatory host milieu may imprint a survival–dysfunction tradeoff from the beginning of cell-product generation.

Exhaustion-associated programming is not confined to the *in vivo* setting. *Ex vivo* manufacturing itself shapes differentiation and can imprint exhaustion-associated features. Prolonged culture drives progressive loss of stem-like and central-memory populations, and shortened manufacturing protocols that limit *ex vivo* expansion yield products with greater proliferative reserve and superior antitumor activity in preclinical models (53). The composition of cytokine support matters similarly: IL-7 and IL-15 preserve less-differentiated phenotypes relative to high-dose IL-2 (54). Activation strength and duration influence the same axis. In addition, many CAR constructs display tonic signaling — antigen-independent signaling arising from clustering or spontaneous aggregation of the CAR itself — which can drive early differentiation and exhaustion-associated phenotypes during manufacturing, before any encounter with tumor antigen (31). The founding state of a CAR-T product is therefore an issue in both modalities, and we do not propose that exhaustion-associated imprinting is unique to *in vivo* generation.

What differs is whether that founding state can be specified, measured, and selected. In the *ex vivo* paradigm, the conditions under which the founding state is established are manufacturing parameters: culture duration, activation modality, γc cytokine composition, and starting-subset selection are all set deliberately, and the resulting product is phenotypically characterized and subject to release criteria before infusion (8, 55). A product whose differentiation profile falls outside specification can, in principle, be rejected or the process revised. The founding state is thus a controlled variable, even when it is imperfectly controlled.

*In vivo*-generated CAR-T cells offer no equivalent control point. The founding state is determined by whichever T cells the vector happens to transduce, in whatever differentiation, activation, and metabolic state those cells occupy at that moment, within the host’s inflammatory and metabolically constrained milieu — under chronic low-level antigen exposure for circulating antigens, hypoxic stress in tissue niches, and TGF-β-rich gradients near tumors. The target population is neither selected nor synchronized; the conditioning environment is the patient rather than a defined medium; no characterization step intervenes between CAR acquisition and function; and there is no product to reject. The costimulatory context in which that state is established is likewise endogenous: the activation and costimulatory signals a T cell has recently received, which are largely APC-derived, shape the differentiation and metabolic state it occupies at the moment of transduction (Section 2.3). Platforms that require or favor activated target cells for efficient transduction are particularly exposed to this dependence, since the same signals that render a T cell permissive to transduction also shape the state it will occupy afterwards. We propose that vector-driven CAR expression during this developmentally vulnerable window establishes a founding-state lock-in — a chromatin program biased toward the fixed dysfunctional state from the earliest days of CAR expression — that subsequent improvements in microenvironment, even effective tumor clearance, may not fully reverse.

Within the framework developed in Section 5.1, this constraint is represented principally through the functional-state construct *η*(*t*), although its effects on subsequent proliferation, death, and persistence may also contribute to the net non-generation term *q_c_*(*t*). Its assignment to *η*(*t*) therefore denotes the dominant functional interpretation rather than an exclusive biological pathway: cells generated under permissive and non-permissive founding conditions may be numerically indistinguishable while differing in proliferative reserve, differentiation state, and effector competence. This has a direct measurement consequence. Because the founding state cannot be specified in advance, it must instead be characterized after the fact, which is why paired baseline and early post-acquisition molecular profiling is listed as an exploratory reporting item in **Table 1** rather than treated as a manufacturing record.

Epistemic status. This concept is hypothesis-generating. The chromatin kinetics summarized above were established in *ex vivo*-transferred T-cell and chronic viral-infection (LCMV) models, both of which differ from the *in vivo* generation context; no published study has directly characterized chromatin landscapes of *in vivo*-generated CAR-T cells at sequential time points. The plastic-to-fixed transition kinetics invoked here are therefore extrapolated, and the proposition that founding-state lock-in materially limits *in vivo* CAR-T function should be treated as a testable hypothesis rather than an established mechanism.

### 2.5 Generation-phase constraint 3: antigen-encounter timing mismatch

The third constraint is the antigen-encounter timing mismatch. In *ex vivo* therapies, the temporal sequence is highly engineered: T cells complete CAR transcription, translation, surface display, and signaling-machinery maturation before encountering tumor antigen. Upon infusion, mature CAR^+^ effector cells engage antigen with stable receptor density and intact downstream signaling.

*In vivo*-generated CAR-T cells face a different sequence with two coupled signaling pressures. First, CAR tonic signaling—well-characterized in *ex vivo* studies as a structure-dependent, antigen-independent activation produced by scFv self-aggregation or 4-1BB-mediated NF-κB activation—is established the moment CAR begins surface expression and can drive premature exhaustion or activation-induced cell death (AICD) even in the absence of antigen (30–32). Second, for high-abundance antigens (BCMA on plasma cells, CD19 on circulating B cells), nascent CAR^+^ T cells encounter cognate antigen during the window when CAR surface density is sub-saturated and co-stimulatory machinery is incomplete. The superposition of tonic and antigen-driven signaling on an immature signaling apparatus may generate activation patterns qualitatively different from those experienced by *ex vivo* CAR-T cells, potentially favoring AICD over productive expansion. One testable expectation is that, in upcoming phase 1 datasets, after adjustment for prespecified disease- and host-level covariates, patients with higher pre-treatment antigen burden at the time of vector administration may show disproportionately blunted peripheral CAR-T expansion relative to lower-burden patients receiving the same vector dose—a deviation that cell-dose-based pharmacokinetic models cannot accommodate.

### 2.6 Disease context shapes vector selection

These layers compound to create a sharp asymmetry between disease contexts that has direct implications for vector design and platform–indication matching. Hematologic targets reside primarily in blood, bone marrow, and secondary lymphoid organs — the same compartments where systemic vector biodistribution peaks and *in situ* generation is most efficient. This spatial co-localization occurs within compartments that are generally more accessible to systemically administered vectors and less structurally restrictive than solid tumors. In solid tumors, by contrast, generation and effector engagement are spatially decoupled: nascent CAR^+^ T cells must traffic across aberrant vasculature, cross dense stroma, and engage immunosuppressive microenvironments (43, 44) while their functional phenotype remains immature and susceptible to founding-state lock-in and AICD.

Disease context should therefore be treated not as a downstream indication label, but as an upstream design variable for vector selection. Hematologic indications primarily require a vector capable of generating a sufficient quantity of CAR^+^ T cells in accessible compartments; solid tumors additionally demand durable expression and sufficient payload bandwidth to co-deliver armoring modules that shield cells through the TME (46, 47). Transient mRNA platforms offer hit-and-run safety profiles that may be particularly favorable for B-cell depletion and immune-reset indications. Aggressive hematologic malignancies may require either repeated dosing or more durable platforms. The persistent onslaught of solid tumors favors durable expression systems, with *in vivo* site-specific integration platforms (such as the TRAC-targeted system by Nyberg et al. (14) representing one particularly promising direction. These divergent disease demands provide the rationale for evaluating *in vivo* CAR-T platforms not as interchangeable delivery vehicles, but as pharmacologically distinct systems with indication-specific strengths and liabilities — a framework developed in Section 3.

## 3 *In vivo* CAR-T platforms: a pharmacological classification

The disease-context analysis of Section 2 establishes that *in vivo* CAR-T platforms must be evaluated not as interchangeable delivery vehicles, but as pharmacologically distinct systems with indication-specific strengths and liabilities. This section develops a four-dimensional pharmacological framework for that evaluation (Section 3.1) and applies it to six platform categories: four functional platform classes (Sections 3.2–3.5), one instructive counterexample (Section 3.6, with detailed mechanistic analysis in **Box 2**), and one frontier paradigm (Section 3.7), closing with platform–indication matching logic (Section 3.8 and **Table 2**); the evidence base for each platform class is summarized in **Supplementary Table 1**.

Box 2 **AAV monotherapy as an instructive counterexample for *in vivo* CAR-T**

 Adeno-associated viruses (AAVs) are the dominant vector class for *in vivo* gene therapy of non-dividing cells (e.g., neurons, hepatocytes, retinal cells). Their application to *in situ* T-cell engineering, however, has consistently encountered vector-determined barriers that highlight the unique kinetic demands of *in vivo* CAR-T pharmacology. These barriers stem from three biological mismatch nodes:

1. Pre-existing humoral immunity. Many natural AAV serotypes are immunogenic in humans, with neutralizing antibody (NAb) seroprevalence varying by serotype and population (56). Pre-existing humoral immunity can reduce productive vector exposure after systemic administration, whereas capsid-specific cellular immune responses may further constrain persistence and redosing (57, 58). Engineered capsids with reduced NAb cross-reactivity (e.g., AAV-hT7 used in Nyberg et al., 2026) (14) partially address this barrier but do not eliminate it.
2. The mitotic dilution effect. Unlike lentiviruses, which integrate into the host genome, AAV genomes primarily persist as episomal concatemers (58). When a successfully transduced T cell encounters its target antigen and undergoes the substantial clonal expansion required for tumor clearance, the episomal AAV DNA is diluted across daughter cells, producing premature loss of CAR expression at the very moment when sustained expression is most therapeutically valuable.
3. Payload constraints. AAVs have a strict ~4.7 kb packaging limit (48). A standard CAR construct, coupled with the requisite T-cell-specific promoters and regulatory elements, substantially constrains the addition of armoring cytokines or safety switches without resorting to dual-AAV strategies, which themselves introduce dose, efficiency, and cost complications.

It is by abandoning AAV as a standalone delivery vehicle and repurposing it as an HDR donor template—paired with transient Cas9-loaded enveloped delivery vehicles as achieved by Nyberg et al. (2026) (14)—that the AAV platform’s role in *in vivo* CAR-T engineering becomes most compelling. This pivot from “AAV as the carrier” to “AAV as the donor” exemplifies a broader principle: platform limitations are most productively understood not as fixed disqualifications, but as cues for repositioning a vector within a multi-component engineering architecture.

**Table 2:** The four-dimensional pharmacological comparison of *in vivo* CAR-T platforms.

Platform class	Generation kinetics (onset and relative magnitude of *p_c_*(*t*))	Duration of CAR expression	Genomic safety	Redosability	Hypothesis-generating indication profile
I. mRNA-LNP	Rapid onset (hours); low-to-moderate magnitude	Transient (days)	Excellent (non-integrating)	Potentially high; platform- and regimen-dependent	Autoimmune disease, fibrosis
II. circRNA-enabled targeted delivery (preclinical)	Demonstrated in a murine model; onset and magnitude not separately characterized	Murine *in vivo*: detectable to ~day 10, baseline by day 14 in one model; not benchmarked against linear mRNA	Potentially favorable (non-integrating payload)	Potentially favorable; therapeutic redosing not characterized	Prospective bounded or repeatable applications; indication matching remains hypothesis-generating
III. Non-viral DNA + transposon (active integration)	Moderate onset; moderate magnitude	Durable	Moderate (semi-random integration)	Moderate	Hematologic malignancy
IV. Targeted LVV	Rapid onset; comparatively high magnitude	Durable	Moderate (semi-random integration)	Currently low (anti-vector immunity)	Aggressive hematologic malignancy (RRMM, LBCL)
V. AAV monotherapy	Low; limited in dividing T cells	Episomal (diluted upon T-cell expansion)	High (non-integrating)	Currently low (pre-existing NAbs)	Instructive counterexample
VI. Site-specific integration (EDV + AAV)	Moderate-to-high magnitude; onset pending characterization	Durable (*TCR*-regulated)	Potentially high (targeted *TRAC* integration)	Pending characterization	Solid tumors (preclinical to date)

This table compares *in vivo* CAR-T platforms across four pharmacological dimensions: generation kinetics, duration of CAR expression, genomic safety, and redosability. These dimensions inform hypothesis-generating platform–indication matching and should be interpreted qualitatively rather than as fixed rankings, because each value depends on dose, vector design, targeting ligand, disease context, and clinical sampling strategy. The entries are platform-level design attributes, not patient-level measurements: the quantities defined in **Box 1** — *p_c_*(*t*), *C*_0_^emergent^, *η*(*t*), and *E*_functional_ — are patient-, disease-, and time-specific and are not platform constants.

Mapping to the production-rate framework (Section 5.1, **Box 1**): Platform-level generation kinetics qualitatively inform the expected magnitude and temporal profile of the latent *de novo* CAR-acquisition rate *p_c_*(*t*), but do not constitute a patient-level measurement of that quantity; duration of CAR expression informs the prespecified operational boundary *τ_g_* and contributes to the CAR/signaling-competence domain of *η*(*t*); genomic safety and redosability together define the platform-specific safety geometry developed in Section 3. No column of this table reports *η*(*t*) itself, which is measured in patients rather than assigned to a platform. Evidence base to date, highest evidence level, principal uncertainty, and principal safety concern for each platform class are summarized in **Supplementary Table 1**. LNP, lipid nanoparticle; LVV, lentiviral vector; AAV, adeno-associated virus; EDV, enveloped delivery vehicle; NAb, neutralizing antibody; RRMM, relapsed/refractory multiple myeloma; LBCL, large B-cell lymphoma; *p_c_*(*t*), latent *de novo* CAR-acquisition rate; *τ_g_*, prespecified operational boundary of the generation phase; *η*(*t*), functional weighting construct (**Box 1**).

### 3.1 A four-dimensional pharmacological framework

To systematically evaluate the rapidly expanding arsenal of *in vivo* delivery vehicles, we propose a four-dimensional framework that shifts the emphasis away from *ex vivo* metrics such as manufacturing scalability and vein-to-vein time toward pharmacologically meaningful dimensions: (i) generation kinetics—the efficiency and speed of *in situ* T-cell reprogramming, shaping the latent *de novo* CAR-acquisition rate *p_c_*(*t*) in **Box 1**; (ii) duration of CAR expression—the half-life of CAR surface expression, which informs the operational boundary *τ_g_* and the therapeutic window; (iii) genomic safety—the spectrum from non-integrating systems to site-specific or semi-random integration; and (iv) redosability—the capacity to administer sequential doses without neutralization by anti-vector immunity. These four dimensions characterize the delivery architecture rather than the receptor it delivers. Receptor-level design choices — binder affinity, hinge and transmembrane composition, and the promoter or genomic context governing expression — further modulate CAR surface density, tonic signaling, and downstream signaling competence (52, 68), and therefore act principally on the CAR/signaling-competence domain of *η*(*t*) largely independently of the delivery platform. Together, these dimensions provide the basis for the platform–indication matching framework summarized in **Figure 3**.

**Figure 3:**
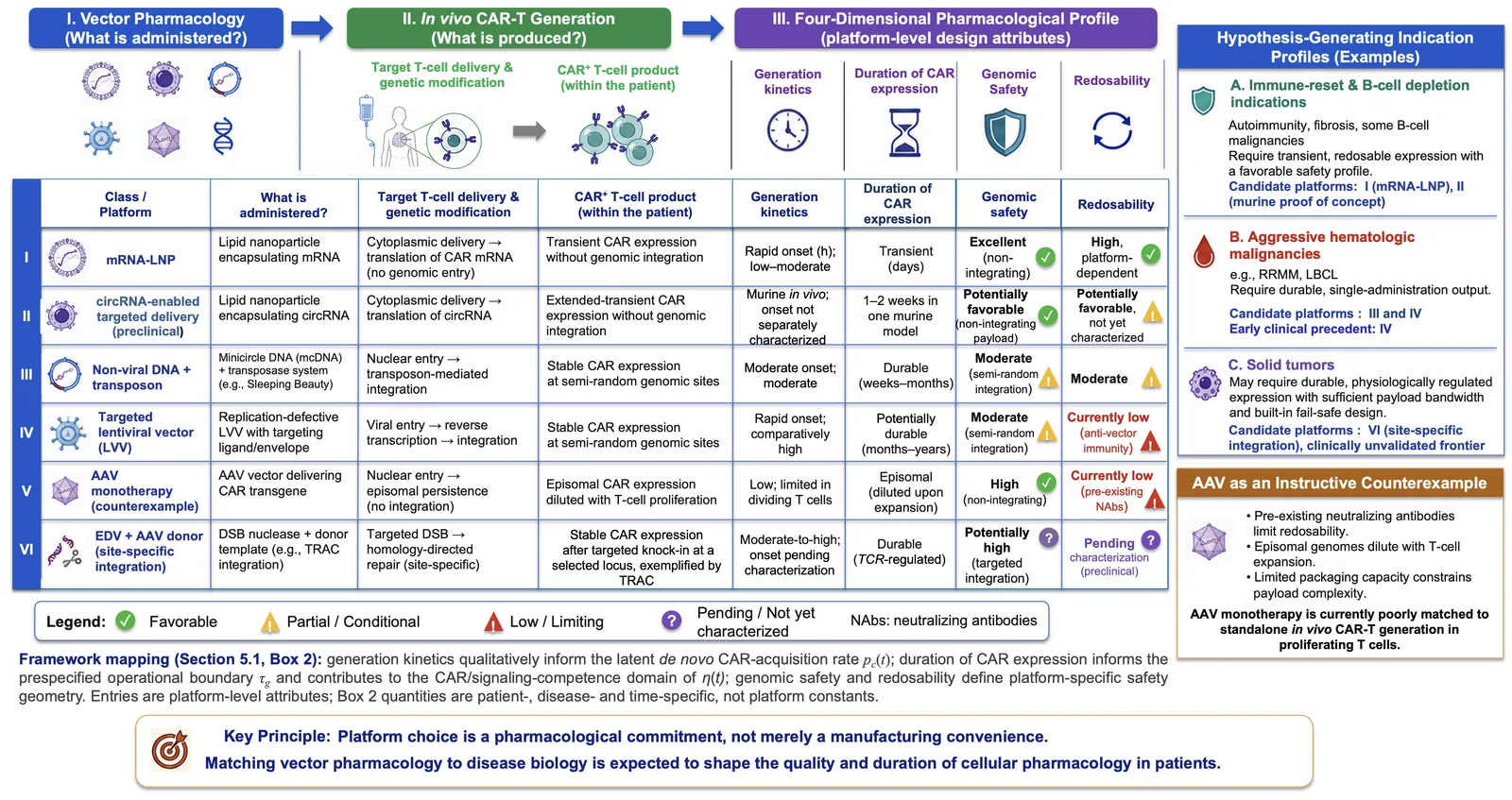
Four-dimensional platform–indication matching for *in vivo* CAR-T therapy. Six *in vivo* CAR-T platform classes are compared across four pharmacologically relevant, platform-level design attributes: generation kinetics, duration of CAR expression, genomic safety, and redosability. These attributes support qualitative, hypothesis-generating platform–indication matching rather than fixed rankings or validated clinical assignments. mRNA–LNP platforms provide rapid, non-integrating, and transient CAR expression and are candidate platforms for indications requiring bounded and potentially repeatable immune-cell activity. Circular RNA represents a candidate payload modality for extending the expression window of transient RNA systems. A single murine study has demonstrated targeted systemic delivery of CAR-encoding circular RNA to endogenous T cells and *in vivo* generation of CAR-expressing T cells (85); the expression kinetics shown were measured in naive mice, whereas extension of CAR-expression duration relative to matched linear mRNA, and therapeutic redosing, remain to be established. Non-viral DNA–transposon systems and targeted lentiviral vectors provide more durable expression but introduce semi-random genomic integration and platform-specific limitations on redosing. AAV monotherapy is shown as an instructive counterexample because pre-existing humoral immunity, episomal dilution during T-cell proliferation, and limited payload capacity restrict its suitability as a standalone CAR-delivery platform. In site-specific integration systems, AAV can instead serve as a donor template paired with transient editing machinery; site specificity is conferred by the combined editing architecture rather than by the AAV donor alone, as exemplified by targeted CAR knock-in at the TRAC locus. The indication profiles shown are qualitative and hypothesis-generating. Transient RNA systems are candidate matches for immune-reset or bounded B-cell-depletion applications. Durable integrating systems may be considered for aggressive hematologic malignancies, although their suitability depends on integration-related risk, redosing limitations, and the required duration of cellular activity. Site-specific integration represents a promising but clinically unvalidated direction for solid tumors that may require durable, physiologically regulated expression, greater engineering flexibility, and built-in fail-safe design. Platform performance and clinical suitability remain dependent on vector design, dose, targeting strategy, host and disease context, and the clinical sampling and analysis window. In the framework developed in Section 5.1 and **Box 1**, generation kinetics qualitatively inform the expected magnitude and temporal profile of the latent *de novo* CAR-acquisition rate *p_c_*(*t*). Duration of CAR expression informs the prospective, platform-specific operational definition of *τ_g_* and contributes to the CAR/signaling-competence domain of *η*(*t*); it does not determine *τ_g_* as an intrinsic biological time point. Genomic safety and redosability together define the platform-specific safety geometry developed in Section 3. The entries shown are platform-level design attributes rather than patient-level measurements: *p_c_*(*t*), *C*_0_^emergent^, *η*(*t*), and *E*_functional_ are patient-, disease-, compartment-, and time-specific quantities and are not platform constants. AAV, adeno-associated virus; CAR, chimeric antigen receptor; DSB, double-strand break; EDV, enveloped delivery vehicle; LBCL, large B-cell lymphoma; LNP, lipid nanoparticle; LVV, lentiviral vector; NAbs, neutralizing antibodies; RRMM, relapsed/refractory multiple myeloma; TRAC, T-cell receptor alpha constant locus.

### 3.2 Class I: mRNA–LNP platforms (transient, redosable)

The trajectory of lipid nanoparticle (LNP)-encapsulated mRNA for *in vivo* CAR-T generation represents the most accelerated translational arc in the field. Early proofs-of-concept by Smith et al. (polymer-based DNA nanocarriers) (59) and Parayath et al. (polymer-based mRNA delivery) (60) established the principle; Rurik et al. then demonstrated CD5-targeted LNP delivery of anti-FAP CAR mRNA (9), achieving cardiac fibrosis reversal in murine models. The development of selective organ targeting (SORT) nanoparticles by Cheng et al. provided the biochemical grammar to shift biodistribution away from the liver toward extrahepatic sites including secondary lymphoid organs (34, 35, 61).

The principal preclinical anchor is the NHP study by Hunter et al., in which a novel ionizable lipid (L829) incorporated into CD8-targeted LNPs substantially mitigated the hepatic biodistribution sink and systemic reactogenicity of earlier formulations (10). The platform achieved robust, T-cell-specific mRNA delivery in cynomolgus macaques, producing what amounts to a transient, hit-and-run pharmacological profile: rapid B-cell depletion across blood, spleen, and lymph nodes followed by reconstitution with a predominantly naive B-cell phenotype. Because mRNA does not integrate and LNP reactogenicity has been clinically managed in mRNA vaccine contexts, this platform offers one of the most favorable genomic-safety profiles and a potentially high degree of redosability among current *in vivo* CAR-T modalities, potentially enabling the CAR-T functional window to be titrated through sequential dosing, subject to platform-specific redosing tolerability and anti-vector immunity.

This platform class has since entered first-in-human evaluation. In a 2026 conference report, seven patients with relapsed or refractory CD19^+^ B-cell lymphoma had been treated with a CD8-targeted lipid nanoparticle encoding a CD19-directed CAR mRNA as of the reported data cutoff, using an initial intravenous administration followed by three boost doses at 3-day intervals; CAR expression peaked within 4–6 hours, near-complete peripheral B-cell depletion was observed between 12 hours and day 3, and no cytokine release syndrome of grade 2 or higher was reported (84). These are observations of *N*_CAR,_*_c_*(*t*) and of pharmacodynamic effect; *p_c_*(*t*) is not separately identifiable from peripheral sampling and no such claim is made here. They nonetheless provide the first human description of the appearance and decay of CAR-positive cells after *in vivo* delivery, and indicate that repeat dosing is compatible with sustained target-cell depletion. The cohort is small, only three patients were evaluable for response, and a full-length peer-reviewed report was not available at our evidence cutoff.

### 3.3 Class II: circRNA-enabled platforms (candidate extended-transient modality)

Circular RNA represents a candidate payload modality for extending the expression window of transient RNA-based *in vivo* CAR-T platforms. Using an engineered permuted intron–exon splicing strategy, Hu et al. generated scarless circular mRNAs encoding CAR constructs and demonstrated greater and more sustained CAR expression in primary human T cells than that achieved with corresponding linear mRNAs (62). CAR expression remained detectable for longer after circular-mRNA electroporation, and the resulting CAR-T cells showed improved antitumor activity relative to linear-mRNA controls without genomic integration. Hu et al. also demonstrated prolonged *in vivo* expression of an LNP-formulated circular-mRNA reporter, supporting the compatibility of this payload format with lipid-nanoparticle delivery. The combination of a circular-RNA payload with a T-cell-targeted delivery system has since been realized: Shi et al. formulated CAR-encoding circular RNA in CD7-targeted lipid nanoparticles and showed that systemic administration generated T cells expressing an EBNA1 chimeric autoantibody receptor — which displays the EBNA1 epitope as bait for anti-EBNA1 B-cell receptors rather than targeting an antigen on the target cell — *in vivo*, selectively depleting EBNA1-specific B cells and attenuating disease in a murine model of experimental autoimmune encephalomyelitis (85). In naive mice given a single intravenous dose, peripheral CAR-positive cells peaked at approximately day 2, remained detectable through day 10, and returned to baseline by day 14 (85). *In vivo* generation by this route is therefore demonstrated rather than prospective, although the evidence remains confined to a single murine model and the expression kinetics were characterized in naive rather than diseased animals. Whether circular RNA extends the duration of CAR expression relative to a matched linear-mRNA formulation, and whether therapeutic redosing is feasible, remain to be established.

### 3.4 Class III: non-viral DNA platforms with active integration (stable, non-viral)

Bridging transient RNA and permanently integrating viruses, non-viral DNA platforms employ transposon-mediated active integration. These should be distinguished from non-integrating S/MAR-based episomal vectors (63), which achieve extended episomal retention through scaffold/matrix attachment region tethering without genomic integration, and align pharmacologically with extended-transient platforms (Class II) rather than the stable-integration platforms here. Although S/MAR vectors have not yet entered human *in vivo* CAR-T trials, their distinctive profile — episomal persistence with lower insertional mutagenesis risk — may complement the existing class structure as the field matures.

The historical anchor for this approach came from the *ex vivo* work by Magnani et al., which provided a preclinical foundation for stable, non-viral CAR integration using the Sleeping Beauty (SB100x) transposition system (64). Bimbo et al. recently translated this principle *in vivo* through the NCtx platform: targeted LNPs functionalized with anti-CD7 and anti-CD3 binders co-encapsulate minicircle DNA (mcDNA) encoding the CAR transgene together with mRNA encoding the SB100x transposase (65). Upon T-cell uptake, the transposase mRNA mediates integration of the CAR transposon into the genome. This Class III platform merges the scalable, low-immunogenicity profile of LNPs with the expression durability of transposon-mediated integration, while introducing semi-random integration as the principal genomic safety consideration.

### 3.5 Class IV: targeted lentiviral vectors (stable, semi-random)

Targeted lentiviral vectors (LVVs) represent the vanguard of stable *in vivo* engineering. The preclinical foundation was established by the VivoVec platform using CD3-targeted lentiviral particles for T-cell reprogramming (36), and subsequently by Nicolai et al., who demonstrated robust CAR-T generation and B-cell depletion in non-human primates using a multidomain fusion ligand displayed on a modified lentiviral envelope (37).

This preclinical scaffolding directly enabled the first-in-human reports of ESO-T01 (11, 12) and KLN-1010 (13). LVV platforms currently provide the clearest clinical evidence for durable *in vivo* CAR-T generation, supporting the deep MRD-negative responses observed in relapsed/refractory multiple myeloma (11, 12). However, these advances come with platform-specific tradeoffs: the semi-random integration profile carries an inherent risk of insertional mutagenesis, and the immunogenicity of the viral envelope — combined with anti-vector neutralizing antibodies — currently constrains redosability, though immune-shielded vectors and alternative envelope pseudotyping strategies are under active development. Targeted LVVs are best understood as “single-administration, durable-output” platforms—a profile well matched to aggressive hematologic malignancies but less suited to indications requiring titration or chronic dosing.

For systemic administration, redosing is not the only unresolved issue: innate immune sensing, complement activation, non-target biodistribution, and where applicable, vector shedding require product-specific characterization (15, 22, 36). Because these properties depend on product design, manufacturing attributes, dose, and route of administration rather than on vector class alone, their magnitude cannot be inferred from the lentiviral platform label and should be evaluated using platform-appropriate biodistribution, shedding, and immunogenicity assays during early clinical development (22).

### 3.6 Class V: AAV monotherapy as an instructive counterexample

Adeno-associated viruses (AAVs) are the dominant vector class in traditional *in vivo* gene therapy for non-dividing target cells. Despite proof-of-concept work (66) and capsid evolution efforts including the Ark313 variant (48, 67), AAV monotherapy has not yet achieved the generation kinetics required for therapeutically relevant *in vivo* CAR-T generation in dividing T cells. **Box 2** provides a full mechanistic breakdown of why AAV monotherapy is poorly matched to standalone *in vivo* CAR-T delivery, despite the platform’s dominance in other gene-therapy applications. As Section 3.7 develops, this same vector class achieves transformative results when repurposed as a homology-directed repair (HDR) donor template rather than as a standalone delivery vehicle.

### 3.7 Class VI: site-specific integration (the frontier paradigm)

To reconcile the simultaneous demands of genomic safety, functional durability, and adequate payload capacity for armoring modules, Nyberg et al. introduced a dual-vector architecture: a CD3-targeted enveloped delivery vehicle (Cas9-EDV) carrying transient CRISPR–Cas9 ribonucleoproteins paired with an evolved AAV capsid (AAV-hT7) delivering a DNA donor template for homology-directed repair-mediated insertion at the endogenous *TRAC* locus (14).

This design exploits a key engineering insight: while neither vector alone is fully T-cell-specific, their intersection is — only cells internalizing both Cas9-EDV and AAV-hT7 undergo CAR knock-in, with the overlap highly enriched for T cells. By placing the CAR transgene under endogenous TCR transcriptional regulation rather than constitutive promoter activity, this approach yields more uniform, physiologically modulated CAR expression and reduced tonic signaling (68)— directly addressing the founding-state lock-in described in Section 2.4 by tempering the very signaling pressures that drive early chromatin remodeling toward exhaustion. In humanized mouse studies, a single intravenous administration generated TRAC-CAR T cells at therapeutic levels and produced complete responses in models of B-cell aplasia, multiple myeloma, and—most notably — a solid sarcoma, representing one of the first reported demonstrations of *in vivo*-generated CAR-T cells achieving complete responses in a solid tumor model (14). Clinical validation in humans remains pending, and questions of HDR efficiency, off-target editing, and dual-vector pharmacokinetics will need to be addressed in IND-enabling studies; nevertheless, Class VI represents one particularly promising direction for translating *in vivo* CAR-T into solid-tumor indications.

### 3.8 Platform–indication matching logic

This 4D framework reveals that no single platform is universally optimal; vector pharmacology must instead be matched to disease pathophysiology. For autoimmune diseases and fibrotic indications, the transient and potentially redosable kinetics of mRNA–LNP platforms may provide a bounded window of B-cell depletion or fibroblast clearance to induce an “immune reset” without the long-term considerations of viral integration (69). CircRNA-enabled targeted delivery could potentially extend this window while retaining a non-integrating payload; *in vivo* generation has now been demonstrated in a murine autoimmune model (85), whereas extension of CAR-expression duration relative to matched linear mRNA, and therapeutic redosing, remain to be established. For aggressive hematologic malignancies such as RRMM and large B-cell lymphoma, the durable, single-administration profile of targeted LVVs aligns with the kinetic demand to outpace tumor proliferation. For solid tumors, transient expression may be insufficient in many settings, whereas semi-random integrating systems raise genomic-safety concerns and still face payload ceilings for multi-cistronic armoring; site-specific integration platforms, by enabling stable expression at a chosen genomic locus while preserving payload bandwidth, emerge as a particularly promising direction—though human clinical validation remains pending.

This indication-stratified logic illustrates the central premise of the production-rate framework: vector platforms are not interchangeable delivery vehicles but pharmacologically distinct systems whose generation kinetics, duration of CAR expression, genomic safety, and redosability collectively determine which clinical applications they can support. The vector-determined engineering constraints that follow from this classification—payload economics and the fail-safe imperative — are developed in Section 4.

## 4 Payload economics and the fail-safe imperative: vector-determined ceilings on cellular therapeutic design

The vector-to-cell pharmacological transition imposes specific engineering constraints that *ex vivo* CAR-T does not face in the same form. Whereas *ex vivo* genetic payloads are constrained primarily by manufacturing complexity, *in situ* generation confronts physical and stoichiometric boundaries dictated by the delivery vector itself — boundaries that cannot be relieved by manufacturing optimization. These vector-determined ceilings shape what CAR-T cells can be built *in vivo*: which armoring modules can be co-delivered, which logic-gated designs can be encoded, and which fail-safe architectures can be installed. This section develops two coupled engineering constraints—payload economics (Section 4.1) and the fail-safe imperative (Section 4.2)—that together define the operational design space for *in vivo* CAR-T.

### 4.1 Vector-specific payload economics

Sophisticated CAR designs increasingly incorporate armoring elements such as secreted pro-inflammatory cytokines, dual-targeted receptors, and Boolean logic gates (39, 46, 47). In the *ex vivo* setting, these larger genetic circuits can be assembled using sequential transductions, electroporated plasmids, or transposon systems, allowing substantially greater design flexibility than is feasible for systemic *in vivo* delivery. *In vivo*, by contrast, they collide with vector-specific payload limits. Lentiviral vectors exhibit a practical packaging ceiling of approximately 8–10 kb, beyond which titer drops sharply. Lipid nanoparticles, despite theoretically larger capacities, face stoichiometric and translational constraints when multiplexing multiple large mRNAs in a single particle (34). AAV vectors face the strictest limit at ~4.7 kb (48), generally precluding packaging of a full-length CAR together with additional armoring modules.

This payload constraint forces a fundamental spatiotemporal tradeoff. Engineering a dual-target or logic-gated CAR *in vivo* requires either encoding all components within a single multi-cistronic vector — which compresses the design budget and, for LVV, decreases viral titer in a length-dependent manner—or distributing the genetic load across multiple vectors. The multi-vector approach, however, relies on stochastic *in vivo* biodistribution. Under the low and heterogeneous multiplicities of infection achievable by systemic delivery, coordinated co-transduction of the same T cell becomes a low-probability, Poisson-like event, rendering multi-vector reprogramming inefficient unless engineered intersection strategies are employed. The dual-vector system of Nyberg et al. (14) exemplifies how this constraint can be turned into a feature: by requiring co-uptake of an enveloped delivery vehicle (carrying Cas9 RNP) and an AAV (HDR donor) for productive editing, the intersection itself confers T-cell specificity that neither vector alone provides.

### 4.2 The fail-safe imperative

Payload economics ultimately collide with a second vector-determined constraint: the fail-safe imperative. In conventional *ex vivo* CAR-T therapy, safety oversight is partly recallable at the product level — clinicians can observe response to an initial dose, monitor adverse events, and withhold subsequent infusions if severe toxicity occurs. *In vivo* CAR-T cell generation substantially erodes this product-level recall capacity. Once the vector is infused, reprogramming and subsequent T-cell expansion proceed autonomously. If off-tumor toxicity, antigen escape, or severe cytokine-release toxicity occurs, the vector itself cannot be recalled, and engineered cells can only be controlled by induced cell death, immunosuppression, or—if available—an engineered fail-safe.

For integrating platforms, passive half-lives are therefore insufficient; controlling toxicity mandates active termination. Since the foundational development of the inducible caspase-9 (iCasp9) system (70) and its first-in-human validation (71), genetically encoded suicide switches have become a staple of advanced cell therapy. For *in vivo* CAR-T employing integrating vectors, a fail-safe is no longer merely an optional enhancement; it is likely to become a central design consideration.

Critically, the fail-safe imperative imposes a direct constraint on payload economics, and this constraint differs fundamentally between integrating and non-integrating platforms. For LVV, incorporating iCasp9 consumes approximately 1.2–1.3 kb of the vector’s packaging budget (70, 71), creating a zero-sum design choice: to fit a fail-safe alongside the core CAR (~2 kb) and essential armoring or co-stimulatory components (~0.5–1 kb), developers must prune other functionalities. The VivoVec platform exemplifies this tension: UB-VV100 encodes a polycistronic payload combining an anti-CD19 4-1BBz CAR with a rapamycin-activated cytokine receptor system, bringing the cassette close to the 8–10 kb practical ceiling and leaving little headroom for additional fail-safe modules (36).

Conversely, for mRNA-LNP platforms, a permanent genetic fail-safe cannot be durably integrated by definition. Safety must rely on intrinsic mRNA degradation, which provides automatic temporal containment but at the cost of requiring repeat dosing to sustain a therapeutic window. Repeat systemic administration of PEGylated LNPs may induce or amplify anti-PEG immunity, creating a potential risk of altered biodistribution, accelerated clearance, and complement-mediated reactions; the clinical magnitude of this effect remains platform- and dosing-regimen-dependent (72). Co-encapsulating a transient suicide-switch mRNA alongside the CAR mRNA does not fully resolve this challenge, because suicide-switch expression kinetics are coupled to the same intrinsic mRNA decay timeline as the CAR itself.

Fail-safe design in *in vivo* CAR-T is therefore not a plug-in module but a platform-level commitment made at the earliest stage of vector design. This coupling between fail-safe architecture and payload budget illustrates a core principle of the vector-to-cell transition: the choice of *in vivo* delivery platform is itself a pharmacological commitment, not a manufacturing convenience — a theme developed further in the living-pharmacokinetics framework of Section 5.

## 5 Living pharmacokinetics: a fifth phase for the *in vivo* generation window

The pharmacological case for treating *in vivo* CAR-T as a category distinct from *ex vivo* CAR-T — built across Sections 2–4 around niche competition, founding-state lock-in, antigen-encounter timing mismatch, payload economics, and the fail-safe imperative — comes to a head when we ask how its kinetics should be modeled, dosed, and clinically interpreted. We argue here that *in vivo* CAR-T does not merely complicate the existing four-phase “living drug” pharmacokinetic framework developed for *ex vivo* products (73–77); it adds an upstream generation phase that current models do not explicitly accommodate. First-in-human datasets accumulated to date should therefore be read with corresponding caution, and the framework should be operationalized — rather than remain rhetorical — through measurable variables and testable predictions (**Box 1**).

### 5.1 An operational scaffold: separating observation, inference, and derivation

The pharmacological difficulty of *in vivo* CAR-T is not simply that its exposure is unknown, but that the administered input and the pharmacologically active entity differ in kind. Vector dose is administered in a defined unit, whereas the active entity is a population of CAR-positive (CAR^+^) T cells whose abundance, functional quality, and spatial distribution evolve over time — and that population does not yet exist at the moment of vector administration. In *ex vivo* CAR-T this gap is closed before dosing: the cellular product is manufactured, characterized, and released, so cell dose is genuinely an input. For *in vivo* CAR-T, the corresponding cellular quantity emerges within the patient and is therefore an outcome.

Before attempting to model this system, an early-phase framework must perform a narrower foundational task: it must specify which quantities are observed, which are inferred, and which are derived, while keeping their dimensions distinct. **Box 1** provides the formal definitions; here we set out the reasoning that assigns each quantity to its respective role.

What is observed. Clinical assays return the abundance or concentration of CAR^+^ T cells in a specified sampled compartment, *N*_CAR,_*_c_*(*t*). This trajectory is the observational primitive for the cellular kinetic quantities in the framework. The apparent net appearance rate,


$$r_{\text{app},c}(t)=\frac{\text{d}N_{\text{CAR},c}(t)}{\text{d}t}$$

is therefore a derived kinetic descriptor rather than a primary observation or a direct measurement of production. It is a compartment-dependent composite of *de novo* CAR acquisition, antigen-driven or homeostatic proliferation, cell death, loss of detectable CAR expression, and trafficking into and out of the sampled compartment, and these contributions cannot be separated by inspection of the peripheral trajectory alone. Treating this apparent net rate as though it were a directly measured production rate would obscure the biological processes embedded within the observation.

What is inferred. The generation-phase biology discussed in Sections 2 and 4 acts through processes that peripheral sampling cannot separately resolve. Conceptually, the observed rate can be decomposed as


$$r_{\text{app},c}(t)=p_{c}(t)+q_{c}(t)$$

where *p*_c_(*t*) denotes the latent *de novo* CAR-acquisition rate and *q*_c_(*t*) collects the net contribution of all non-generation processes, including proliferation, cell death, loss of detectable CAR expression, and compartmental exchange. Only their sum is identified by the observed cellular trajectory; this is therefore a conceptual decomposition, not an estimable two-component model, unless orthogonal measurements are introduced.

This distinction also anchors the name of the framework. The “production rate” in production-rate pharmacology refers to *p*_c_(*t*), the latent rate at which cells newly acquire detectable CAR expression, rather than to the apparent rate returned by longitudinal sampling. Vector design may shape the magnitude and duration of *p*_c_(*t*), but its independent value cannot be recovered from peripheral cellular kinetics alone.

Where the generation-phase constraints act. The constraint classes developed in Sections 2 and 4 have different primary loci within this structure. These assignments identify dominant pharmacological consequences rather than exclusive mathematical pathways, and none is assumed to be independently identifiable from peripheral kinetics.

Delivery attrition (Section 2.1), together with the payload constraints discussed in Section 4, acts primarily on the achievable magnitude and duration of *p*_c_(*t*). Biodistribution, target-cell tropism, transduction or editing efficiency, and cargo delivery jointly determine how many cells newly acquire detectable CAR expression per unit time. Payload and construct design may in addition influence the functional properties subsequently represented through *η*(*t*).

Niche competition in the absence of lymphodepletion (Section 2.3) acts primarily through *q*_c_(*t*). Newly generated CAR^+^ cells enter an intact endogenous T-cell pool and may face constraints on homeostatic cytokine support, space, proliferation, and persistence. Similar generation inputs can therefore give rise to different observed *N*_CAR,_*_c_*(*t*) trajectories, and distinguishing these possibilities requires orthogonal measurements rather than inspection of peripheral kinetics alone.

Founding-state lock-in (Section 2.4) and antigen-encounter timing mismatch (Section 2.5) are represented primarily through the functional-state construct *η*(*t*). Cells that acquire CAR without controlled activation or costimulatory conditioning, or that encounter antigen before adequate CAR expression is established, may be numerically similar to cells that do not, yet differ in proliferative reserve, differentiation state, metabolic fitness, and effector competence. These processes may also alter subsequent proliferation, death, and persistence and thus contribute to *q*_c_(*t*). This is the circumstance in which cell number alone may be insufficient, and it motivates evaluation of context-specific functional measurements alongside cellular kinetics.

Host determinants do not constitute a separate component of this framework but act across all three positions. Baseline T-cell composition and target-cell availability constrain *p*_c_(*t*), as do host determinants of vector disposition — including mononuclear phagocyte activity and pre-existing or emergent anti-vector immunity — which reduce the productive vector available for transduction; immune-mediated elimination of already generated CAR-positive cells, whether directed against vector-derived antigens or against the CAR itself, contributes instead to *q_c_*(*t*). The endogenous lymphoid niche and homeostatic cytokine availability act principally through the post-acquisition processes collected in *q_c_*(*t*). Antigen burden, antigen-presenting-cell availability, and the costimulatory signals present around CAR acquisition shape *η*(*t*), while continued costimulatory and cytokine support also acts on *q*_c_(*t*) (Section 2.3). Representing the host as an additional multiplicative term would duplicate effects already carried by these components and would introduce a quantity that is not separately identifiable from peripheral kinetics. Host status is therefore treated as a set of measurable covariates of the existing components — specified as core reporting items in **Table 1** and as candidate predictors in Prediction 1 — rather than as a fourth axis of the framework.

Why the mapping matters. This assignment is not bookkeeping. It implies that interventions acting at different positions are not necessarily interchangeable. Vector engineering primarily shapes *p*_c_(*t*); conditioning strategies and cytokine support may alter both target-cell availability and the post-acquisition processes collected in *q*_c_(*t*); antigen timing and design features that influence the founding state are evaluated primarily through the functional measurements represented by *η*(*t*). Collapsing these effects into a single scalar potency measure would obscure pharmacological axes that can be manipulated separately and that fail through different mechanisms.

The same observed outcome can consequently arise through distinct biological routes. A low *C*_0_^emergent^, for example, may reflect limited *de novo* CAR acquisition, insufficient proliferation, increased cell loss, loss of detectable CAR expression, trafficking out of the sampled compartment, or combinations of these. These alternatives cannot be distinguished from the cellular trajectory alone and require different supporting measurements. Conversely, similar values of *C*_0_^emergent^ may conceal important differences in functional state and downstream therapeutic potential.

The generation-to-cellular-kinetics handoff. At the prespecified operational boundary *τ*_g_,


$$C_{0}^{\text{emergent}}=N_{\text{CAR},c}(\tau_{g})$$

provides a compartment-specific cellular reference for linking the generation phase to downstream analyses of distribution, expansion, contraction, and persistence. *τ_g_* is a platform-specific analytical convention, not a universal biological point at which generation abruptly stops; generation and downstream expansion may overlap. Its definition should therefore be justified prospectively using platform-appropriate vector, transgene, and cellular kinetic measurements rather than selected *post hoc* by curve fitting.

The derived exposure quantity. Cellular exposure is defined on the state variable *N*_CAR,_*_c_*(*t*) rather than on an appearance rate, through the candidate functionally weighted exposure metric


$$E_{\text{functional}}=\int_{0}^{T}N_{\text{CAR},c}(t)\;\eta (t)\;\text{d}t$$

as described in **Box 1**. This construction is analogous to a concentration–time AUC when concentration is used as the cellular state variable, while additionally incorporating a time-dependent functional weighting construct. **Figure 4** summarizes this three-layer pharmacokinetic architecture, linking the administered vector input to the generation and early-selection phase and, through the emergent cellular boundary condition *C*_0_^emergent^, to downstream cellular kinetics and the candidate functionally weighted exposure *E*_functional_.

**Figure 4:**
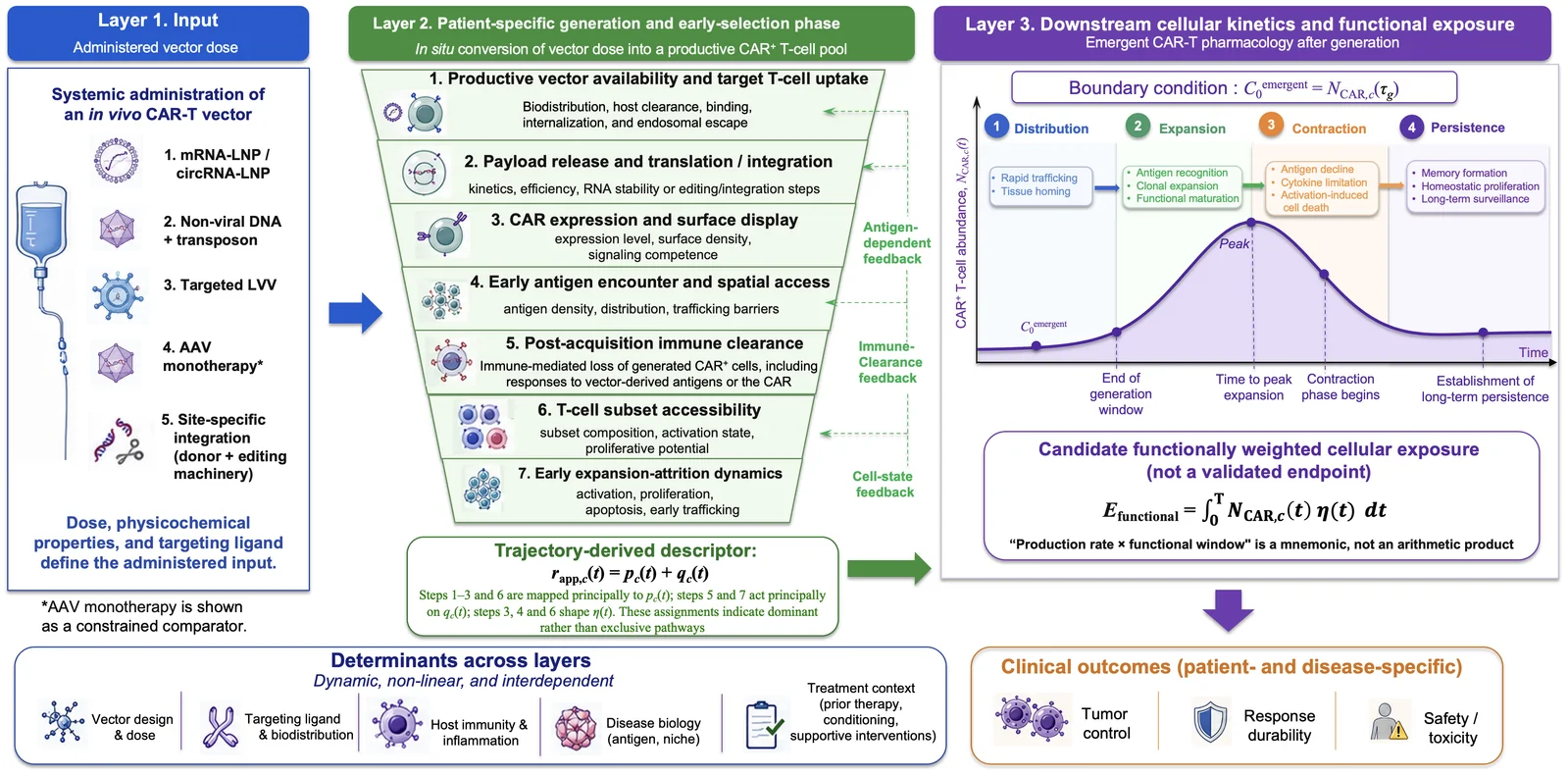
A three-layer pharmacokinetic funnel for *in vivo* CAR-T generation. Layer 1, administered input: a systemically delivered vector dose defined by platform, physicochemical properties, targeting-ligand configuration, and payload. Layer 2, generation and early-selection phase: patient- and platform-dependent conversion of vector input into a productive CAR^+^ T-cell pool through target T-cell uptake, payload release and expression or integration, CAR surface display, early antigen encounter, immune clearance, T-cell subset accessibility, proliferation, trafficking, and attrition. The observational primitive of this layer is the longitudinal CAR-positive T-cell abundance in a specified sampled compartment, *N*_CAR,_*_c_*(*t*), from which the apparent net appearance rate *r*_app,_*_c_*(*t*) is derived. Because *r*_app,_*_c_*(*t*) is a net derivative, it is a compartment-dependent composite of *de novo* CAR acquisition, proliferation, cell death, loss of detectable CAR expression, and trafficking, and should not be interpreted as a direct measure of biological production. Conceptually it decomposes as *r*_app,_*_c_*(*t*) = *p_c_*(*t*) + *q_c_*(*t*), where *p_c_*(*t*) is the latent *de novo* CAR-acquisition rate and *q_c_*(*t*) the net contribution of all non-generation processes; only their sum is identified by the observed trajectory. Within this structure, steps 1 — including innate sensing and anti-vector immunity when these reduce productive vector availability — together with steps 2, 3 and 6 act principally on *p_c_*(*t*), step 5 is restricted to immune-mediated clearance of already generated CAR^+^ cells and, together with step 7, acts principally on *q_c_*(*t*). Steps 3, 4, and 6 shape the functional-state weighting construct *η*(*t*). These assignments identify dominant interpretations rather than exclusive pathways. Layer 3, downstream cellular pharmacology: the emergent cellular boundary condition *C*_0_^emergent^ = *N*_CAR,_*_c_*(*τ_g_*) — the observed CAR-positive T-cell abundance at a prespecified, platform-specific operational boundary *τ*_g_ rather than at a universal biological time point — enters the canonical distribution, expansion, contraction, and persistence trajectory. The candidate functionally weighted cellular exposure, *E*_functional_ = ∫_0_^T^ *N*_CAR,_*_c_*(*t*)·*η*(*t*) d*t*, integrates CAR-positive T-cell abundance over a prespecified analysis horizon *T* with a time-dependent functional-state weighting construct, *η*(*t*), thereby distinguishing persistent but dysfunctional cells from shorter-lived, functionally competent populations. *E*_functional_ is advanced as a candidate pharmacological exposure metric rather than a validated endpoint, and “production rate × functional window” is retained only as a heuristic mnemonic for the two axes concerned, not as an arithmetic product (**Box 1**). Vector design and dose, biodistribution, host immunity, disease biology, treatment context, and cellular-state feedback interact non-linearly across layers to shape disease control, response durability, and safety. Dashed arrows denote proposed feedback relationships rather than established quantitative dependencies. AAV, adeno-associated virus; CAR, chimeric antigen receptor; LNP, lipid nanoparticle; LVV, lentiviral vector; *C*_0_^emergent^, emergent cellular boundary condition; *E*_functional_, candidate functionally weighted cellular exposure; *N*_CAR,_*_c_*(*t*), CAR-positive T-cell abundance in sampled compartment *c*; *p_c_*(*t*), latent *de novo* CAR-acquisition rate; *q_c_*(*t*), net non-generation term; *r*_app,_*_c_*(*t*), apparent net appearance rate; *T*, prespecified analysis horizon; *η*(*t*), functional weighting construct; *τ_g_*, prespecified operational boundary of the generation phase.

Two constraints follow. First, cellular exposure cannot be constructed by integrating an apparent net appearance rate, weighted or otherwise: the time integral of *r*_app,_*_c_*(*t*) yields the net change in observed cellular abundance over the integration interval, not an abundance–time exposure. Integrating a rate and integrating a cellular state therefore produce dimensionally and biologically distinct quantities. Second, no universal *η*(*t*) is assumed. Functional state is platform-, disease-, target-, compartment-, and time-specific, and, as set out in **Box 1**, is at present better reported through prespecified domain-specific measurements than collapsed into a single scalar weighting rule.

What this framework is not. The production-rate framework is an operational scaffold and testable organizing hypothesis, not a validated population pharmacokinetic model. It does not claim that the latent components *p*_c_(*t*) and *q*_c_(*t*) are separately identifiable from currently available peripheral datasets, and it proposes no fitted values for them. Nor does it assert that *E*_functional_ is a validated clinical endpoint. Rather, *E*_functional_ is a candidate pharmacological exposure metric whose construction can be prespecified and reported, and whose incremental predictive value relative to vector dose, cellular *C*_max_, and unweighted cellular exposure can be tested empirically.

“Production rate × functional window” is retained only as a mnemonic for two axes along which *in vivo* CAR-T departs from conventional cell-dose pharmacology: the kinetics and duration of cellular generation, and the functional quality of the resulting cells. The multiplication sign denotes joint conceptual dependence, not dimensional equivalence or an arithmetic product. The resulting claim is deliberately limited and empirically testable: **Box 1** states four falsifiable predictions, and **Table 1** specifies the measurements required to evaluate them.

### 5.2 Evidence from first-in-human trials: reading the signals carefully

Five key translational reports accumulated across 2025–2026 — one landmark non-human primate study and four early first-in-human reports from three distinct *in vivo* CAR-T programs, of which two are successive reports from the ESO-T01 development program rather than independent platform validations — give a first opportunity to test these predictions and to confront the limits of preclinical kinetic models when translated to humans. Each warrants careful reading given the small sample sizes (no more than seven patients per report), single-arm designs, and limited follow-up at the time of publication.

The NHP validation by Hunter et al. (10) remains the cleanest preclinical anchor, showing dose-dependent B-cell depletion in cynomolgus macaques after CD8-targeted L829-tLNP administration of anti-CD20 CAR mRNA. The dose-ranging data are consistent with, although they do not prove, a generation-phase bottleneck—precisely what a *p_c_*(*t*) limited by receptor-mediated uptake and translational capacity would predict.

In humans, the case series of Xu et al. (11) in four heavily pretreated patients with relapsed/refractory multiple myeloma (RRMM), using ESO-T01 (a nanobody-targeted, immune-shielded lentiviral vector encoding an anti-BCMA CAR), demonstrated feasibility of a single intravenous infusion without leukapheresis or lymphodepletion. The completed phase 1 trial by An et al. (12) extended this to five patients and reported objective responses in 4 of 5 patients, including three stringent complete remissions, with MRD negativity in all four evaluable responders at 10^-5^ sensitivity by day 60. Safety, the primary endpoint, was less reassuring: all five patients experienced grade 3 or higher adverse events, cytokine release syndrome occurred in four, and one patient died of extramedullary disease-related spinal cord compression, attributed by the investigators to underlying myeloma rather than to treatment. The trial was stopped early in 2025 after five patients, limiting sample size and follow-up and leaving the field with a limited human dataset (12). The translational relationship between vector dose, *in situ* generation, and clinical signal therefore remains incompletely defined. In parallel, the inMMyCAR study of KLN-1010 (13) has reported preliminary signals of activity in early dose escalation in RRMM, providing an independent test of whether the lentiviral *in vivo* CAR-T mechanism generalizes across vector designs — though the evidence base remains three patients in a conference abstract with short follow-up. A third program has entered the clinic on a non-integrating platform: the CD8-targeted LNP first-in-human report describes rapid CAR appearance and near-complete B-cell depletion within days in seven patients treated as of the data cutoff, but with only three patients evaluable for response and no full-length report available (84). Its kinetic profile — appearance within hours rather than days — differs qualitatively from the lentiviral programs, consistent with the platform-level distinction drawn in Section 3.

Two patterns emerge across these datasets. First, where peripheral CAR^+^ T-cell expansion has been measured, the kinetic profile appears recognizable as the canonical phases of expansion, contraction, and persistence familiar from *ex vivo* products (11, 12). This suggests that once functional CAR-T cells are successfully generated *in situ*, their observed peripheral kinetics may resemble the canonical phases described after *ex vivo* CAR-T-cell administration. Second, however, the magnitude and timing of peak CAR^+^ T-cell counts (cellular *C*_max_) vary substantially among patients receiving the same vector dose, in ways that vector dose alone does not predict. This is what the *in situ* generation-phase framework anticipates: the canonical downstream phases may remain broadly recognizable once a detectable CAR-positive T-cell population has emerged, but the upstream conversion of vector dose into the emergent *C*_0_^emergent^=*N*_CAR,_*_c_*(*τ_g_*) appears patient-specific and remains incompletely characterized. More broadly, generation-phase variance has implications beyond statistical inference. In early-phase trials with small cohorts, large inter-patient variability in cellular *C*_max_ at a fixed vector dose can widen confidence intervals around efficacy estimates, complicate dose selection and benefit–risk assessment, and increase the uncertainty faced by long-horizon development programs. The production-rate framework is therefore not only a scientific lens but also a translational risk-management lens for the field.

These observations collectively represent early proof-of-clinical-feasibility rather than confirmed efficacy. The kinetic patterns hint at — but cannot yet confirm — the structural separation between administered input (vector dose) and functionally weighted cellular exposure (*E*_functional_, **Box 1**) that our framework predicts. The next wave of first-in-human datasets — larger cohorts, more uniform sampling, and pharmacodynamic measurements designed to capture *N*_CAR,_*_c_*(*t*) and *η*(*t*) (**Box 1**)—will determine whether this separation holds.

### 5.3 Antigen burden: a shared determinant acting at a different position

Antigen burden is not unique to *in vivo* CAR-T. In *ex vivo* CAR-T therapy, pretreatment tumor burden—an imperfect clinical proxy for antigen burden, because antigen-bearing normal cells may also contribute and antigen density per cell is not captured—has been associated with cellular expansion, efficacy, and toxicity (78, 79), and the magnitude of CAR-T-cell expansion has itself been associated with both clinical response and cytokine release syndrome severity (73). In the ZUMA-1 analysis of axicabtagene ciloleucel in large B-cell lymphoma, durable response was associated more strongly with rapid CAR-T-cell expansion commensurate with pretreatment tumor burden than with tumor burden or expansion considered separately; product T-cell fitness and host systemic inflammation were also important correlates (78). In a separate cohort of adults receiving CD19-directed CAR-T-cell therapy, high marrow tumor burden was identified as an independent baseline predictor of severe cytokine release syndrome (79). Chronic antigen stimulation can in turn promote exhaustion-associated dysfunction (8, 25, 26). Notably, the sets of factors associated with durable response and severe toxicity were not identical (78), a distinction that Prediction 4 extends to the *in vivo* setting. These relationships are product- and disease-specific, and the extent to which they generalize across constructs, diseases, and treatment contexts remains uncertain. Accordingly, treating antigen burden as a problem unique to *in vivo* CAR-T would mischaracterize the field.

What differs is where in the pharmacological sequence antigen burden acts. In the *ex vivo* paradigm, burden acts on a product that has already been manufactured, characterized, and released. The founding state is fixed before the cells meet antigen, and burden therefore modulates what happens to a defined cellular input: it shapes expansion, persistence, and the rate at which chronic stimulation drives exhaustion. In the terms of Section 5.1, burden in that setting is a determinant of the trajectory downstream of a known and characterized cellular input.

For *in vivo* CAR-T, antigen burden is present during generation itself, and therefore acts at two positions that the *ex vivo* sequence does not distinguish. Through the antigen-encounter timing mismatch described in Section 2.5, high-abundance antigen is engaged while CAR surface density is still sub-saturated and the signaling apparatus is incomplete, shaping the functional quality of cells at the moment their founding state is being established; this contribution is represented through *η*(*t*). Separately, activation-driven proliferation and activation-induced cell death alter the number of CAR^+^ cells retained in the sampled compartment; this contribution is represented through *q_c_*(*t*). Antigen burden may in addition influence the activation state, and hence the transduction permissiveness, of target T cells before CAR acquisition, in which case a contribution to *p_c_*(*t*) cannot be excluded.

The practical consequence is that, for *in vivo* CAR-T, antigen burden is not merely a covariate of response, as it is in the *ex vivo* setting; it is also a covariate of the emergent product itself. Two patients with identical vector doses but different antigen burdens may generate CAR^+^ populations that differ in both number and functional state, and these two differences are not interchangeable. This is why antigen or disease burden at vector administration is specified as a core reporting item in **Table 1** and as a candidate generation-phase covariate in Prediction 1, and why the testable expectation stated in Section 2.5 — that, after adjustment for prespecified disease- and host-level covariates, higher pre-treatment antigen burden may be associated with disproportionately attenuated peripheral expansion at a fixed vector dose — is a prediction about the generation phase rather than a restatement of the *ex vivo* burden–response relationship.

Antigen burden thus illustrates the general point of Section 5.1. Several determinants of CAR-T pharmacology are shared between the two modalities; what the production-rate framework contributes is not a claim that they are new, but a structure that specifies where each acts and therefore what would have to be measured to distinguish their contributions.

### 5.4 The dose–response paradox

The absence of a reliable dose–response relationship is not unique to *in vivo* CAR-T; it has been a recognized feature of CAR-T pharmacology since the earliest approvals (80). In June 2025, the FDA eliminated the REMS requirements for currently approved CD19- and BCMA-directed autologous CAR-T products, while retaining routine pharmacovigilance and long-term postmarketing safety follow-up requirements (81). Population PK and exposure–response analyses have been used to inform dose selection, but exposure–response relationships are typically flat or non-monotonic across the dosing range, and remain product-specific rather than class-wide (82).

*In vivo* CAR-T adds a further layer of non-linearity. The “input” is no longer a defined cell product but a vector dose; the relevant intermediate variable is the cellular *C*_0_^emergent^ (**Box 1**) — itself the emergent output of the *in situ* generation phase — gated by patient-specific generation-phase efficiency; and the “output” is peripheral CAR^+^ T-cell expansion and clinical response. At each transition node — vector dose → *C*_0_^emergent^, and *C*_0_^emergent^ → cellular *C*_max_ or cellular exposure — the relationship is non-linear, saturable, and modulated by host factors that vector dose alone cannot capture. The practical consequence is that the regulatory dose–response framework that has, with difficulty, accommodated *ex vivo* CAR-T may not seamlessly extend to *in vivo* CAR-T without explicit revision — a challenge developed further in Section 6.

We close this section with a note of first-in-human caution. The translational arc from Rurik (9) to Nyberg (14) is genuinely remarkable. However, the early termination of the ESO-T01 trial after five patients, the substantial variability of peripheral CAR-T expansion at fixed vector dose in early human cohorts, and the FDA’s continued documentation of dose–response paradoxes for the better-characterized *ex vivo* class, collectively suggest that the field is not yet positioned to confidently predict human pharmacology from preclinical *in vivo* CAR-T datasets.

**Box 1** translates this caution into a testable reporting framework. It defines the generation-phase variables that next-generation *in vivo* CAR-T trials should measure — not as a validated PK model, but as a scaffolding for organizing the variables, the feedback loops, and the early clinical signals that the next wave of first-in-human studies must report with kinetic, not solely clinical, sensitivity. The four falsifiable predictions stated there, and the measurements specified in **Table 1**, define what would confirm, refine, or falsify the framework as data accumulate.

It is worth stating plainly how far existing datasets can carry this evaluation, since a framework that could only be tested by studies not yet designed would be of limited immediate value. Published *in vivo* CAR-T datasets report longitudinal CAR^+^ T-cell measurements and cellular *C*_max_, so published datasets can provide supporting evidence for Prediction 1 by documenting within-dose interpatient variability. They cannot test the primary prediction, which requires out-of-sample comparison of dose-only and prespecified multivariable models across dose levels. Reconstruction of *r*_app,_*_c_*(*t*) is constrained by sampling density; anchoring *τ_g_* requires vector and transgene kinetics that are not consistently reported; and functional-state domains are largely unreported, so that *E*_functional_ and the domain-specific exposures *E_j_* cannot presently be computed for any published cohort. The immediate obstacle to evaluating the framework is therefore not conceptual but a reporting gap, and it is a gap that the checklist in **Table 1** is designed to close prospectively rather than retrospectively.

## 6 Vector-determined translation: manufacturing, quality control, and regulatory logic

The pharmacological reframing developed in Sections 1–5 has practical consequences beyond vector design. If *in vivo* CAR-T activity depends on generation kinetics rather than on infused cell dose, scaffolded by an *in situ* generation phase, and constrained by vector-determined safety geometry, then its manufacturing requirements, quality-control logic, and regulatory pathway cannot be inherited unchanged from *ex vivo* CAR-T. The central operational question is therefore: what does it mean to develop and regulate a therapy in which the vector — not a pre-manufactured cell product — is the entity that can be characterized, released, and dosed before administration?

### 6.1 Vector-centric manufacturing and quality control

The most immediately compelling case for *in vivo* CAR-T is what it eliminates. The *ex vivo* paradigm requires patient-specific leukapheresis, multi-day GMP manufacturing of a personalized living cell product, cryopreservation, chain-of-custody management, and vein-to-vein intervals that can extend to several weeks (3). *In situ* generation can potentially circumvent these bottlenecks: an off-the-shelf vector product can be manufactured, characterized, and released under platform-specific CMC requirements, without patient-specific cell manufacturing.

This logistical simplification, however, creates an analytical inversion that remains underappreciated. Three challenges follow. First, the final cellular drug product does not exist prior to administration; it is generated *in situ* within each patient. Conventional release testing of the CAR^+^ T-cell product is therefore impossible — what can be tested and released is the vector, not the cells it will produce. Second, vector batch-to-batch consistency must carry much of the analytical burden that *ex vivo* cellular release testing previously absorbed (21). Variations in vector titre, empty-to-full capsid ratio, infectious-to-physical particle ratio, LNP encapsulation efficiency, RNA integrity, targeting-ligand density, or residual impurities may translate directly into patient-level variability during the generation phase. Third, traditional cellular critical quality attributes — CAR^+^ frequency, CD4/CD8 ratio, memory phenotype, integration copy number, and potency — become post-administration pharmacodynamic observations rather than pre-release specifications. They can no longer be used to withhold a substandard cellular product before infusion.

The result is a shift from cell-product release testing to vector-centric quality control coupled with real-time pharmacodynamic monitoring of the *in situ*-generated cell population. For developers of gene and cell therapies, this is not a peripheral regulatory issue but a core pharmacological consequence: vector attributes become upstream determinants of cellular exposure.

### 6.2 Regulatory classification follows vector architecture

CAR-T cells are currently regulated within gene-therapy frameworks (20, 21). In these frameworks, post-treatment monitoring and long-term follow-up requirements are influenced strongly by vector type, genomic persistence, biodistribution, and potential for delayed adverse events. Conventional *ex vivo* CAR-T products, manufactured with integrating lentiviral or retroviral vectors, have fit relatively cleanly into long-term follow-up pathways designed to monitor insertional mutagenesis and delayed toxicities (22).

*In vivo* CAR-T fragments this alignment. The platform classes reviewed in Section 3 occupy different positions across the spectrum of persistence and genomic risk. Integrating lentiviral vectors, including targeted LVV platforms, inherit the concerns associated with durable semi-random integration. AAV-enabled site-specific integration platforms introduce a different regulatory logic: the delivery vehicle may be transient, but the intended genomic edit is permanent (14). mRNA-LNP platforms, by contrast, are non-integrating and produce transient CAR expression, placing them in a less clearly defined regulatory space for *in situ*-generated cell therapies. Non-viral DNA platforms relying on transposon integration occupy another hybrid category, combining non-viral delivery with durable genomic insertion.

This platform-dependent fragmentation has practical consequences. A single therapeutic concept—*in vivo* generation of CAR^+^ T cells—may require different CMC standards, biodistribution studies, genotoxicity assessments, redosing strategies, pharmacovigilance windows, and risk-management plans depending on vector chemistry and persistence. The June 2025 lifting of Risk Evaluation and Mitigation Strategy requirements for conventional *ex vivo* CAR-T products (81) reflects accumulated experience with that class; *in vivo* CAR-T, with its highly variable generation-phase kinetics, may prompt renewed risk-management requirements depending on platform persistence, tissue biodistribution, and toxicity profile. Although developed primarily for *ex vivo*-manufactured CAR-T products, existing FDA guidance establishes relevant expectations for CMC, pharmacology and toxicology, clinical study design, and long-term safety evaluation (83). How these expectations should be adapted to vector-administered *in vivo* CAR-T products remains platform-specific and will require regulatory clarification. Transient platforms should not be overburdened by monitoring frameworks designed for durable integration, whereas integrating platforms should not be under-monitored simply because the cellular product is generated *in vivo* rather than manufactured *ex vivo*. Recognizing *in vivo* CAR-T as a vector-determined pharmacological category is therefore a prerequisite for aligning regulatory oversight with biological risk.

### 6.3 Testing the production-rate framework in early clinical datasets

The production-rate framework makes a practical demand on emerging clinical studies: vector dose should not be treated as a sufficient proxy for cellular exposure. Whereas Section 5.4 considered how far already-published datasets can carry this evaluation, the requirement set out here is prospective. As phase 1/2 datasets mature, *in vivo* CAR-T pharmacokinetics should be evaluated by asking whether variability in CAR^+^ T-cell expansion, persistence, and function is better predicted by generation-phase variables than by vector dose alone, and whether that improvement holds under out-of-sample evaluation rather than in-sample fit.

Operationally, this requires pharmacodynamic sampling that captures not only peripheral CAR^+^ cell frequency or cellular *C*_max_, but also the longitudinal cellular trajectory *N*_CAR,_*_c_*(*t*) from which the apparent appearance rate is derived, durability of CAR expression, functional phenotype, antigen burden at administration, baseline T-cell composition, anti-vector immunity, and early attrition. These are the variables required to anchor a prespecified *τ*_g_, to compute the emergent boundary condition *C*_0_^emergent^, and — where functional-state domains are measured — the candidate exposure metric *E*_functional_ or its domain-specific counterparts, as defined in **Box 1**; the corresponding reporting requirements are set out in **Table 1**. Continuation of KLN-1010 dose-escalation studies (13), follow-on programs in the ESO-T01 lineage (12), and emerging *in vivo* CAR-T programs for solid tumors and preclinical site-specific engineering approaches (14, 15, 18) will provide opportunities to test whether prespecified variables improve out-of-sample prediction of interpatient cellular pharmacokinetic outcomes beyond vector dose alone.

Conventional rule-based dose-escalation designs, such as the 3 + 3 design, are transparent and operationally simple, but decisions are driven primarily by dose-limiting toxicities observed in small cohorts and do not formally use accumulating cellular pharmacodynamic information. Model-assisted or model-based adaptive approaches can instead update dose–toxicity estimates as data accrue and, where prespecified and supported by adequate sampling, can incorporate pharmacodynamic evidence of biological activity (82). For *in vivo* CAR-T, a pragmatic approach would be safety-constrained escalation in which dose-limiting toxicity and predefined organ-specific or inflammatory safety signals govern escalation and de-escalation, while early vector and transgene kinetics, *N*_CAR,_*_c_*(*t*), *C*_0_^emergent^, cellular *C*_max_, target-cell depletion, and anti-vector immunity inform cohort expansion, schedule refinement, and selection among doses with acceptable safety (83). The latent components *p_c_*(*t*) and *q_c_*(*t*), and the currently unvalidated *E*_functional_, should not serve as standalone dose-assignment variables. Any pharmacodynamic thresholds, analysis windows, adaptation rules, and stopping criteria should be specified prospectively and evaluated through design simulations rather than introduced after inspection of trial outcomes.

If vector dose alone proves sufficient to anchor *in vivo* CAR-T pharmacokinetics, then the production-rate framework would need to be reconsidered, as stated in Prediction 1 (**Box 1**). If, however, prespecified generation-phase variables consistently improve out-of-sample prediction of cellular exposure and clinical activity relative to vector dose alone, the implication would be structural: *in vivo* CAR-T should be recognized as a distinct vector-determined pharmacological category, governed by its own dosing logic, platform-specific safety geometry, and regulatory pathway. The shift from cell-dose to production-rate pharmacology is therefore not only a conceptual reframing, but also an operational framework for developing *in vivo* cell therapies.

## 7 Concluding remarks

The translational arc reviewed here — from murine proof-of-concept to first-in-human reports and emerging site-specific *in vivo* T-cell engineering — is advancing faster than the conceptual language used to interpret it. The central argument of this Review is that *in vivo* CAR-T cell generation is not simply an “in-body” version of *ex vivo* CAR-T, but a vector-to-cell pharmacological transition in which vector pharmacology directly shapes cellular pharmacology. Across platforms, the vector is not merely a delivery vehicle: its biodistribution, payload economics, integration architecture, fail-safe design, and redosability collectively shape the rate, durability, and functional quality of the CAR-T cells it generates.

This reframing supports a testable organizing hypothesis rather than a fixed operational rule: that prespecified generation-phase variables will improve out-of-sample prediction of cellular exposure and clinical activity relative to nominal vector dose alone. “Production rate × functional window” is retained throughout as a mnemonic for the two axes concerned, not as an arithmetic product. **Box 1** distinguishes the observed cellular trajectory *N*_CAR,c_(*t*), the emergent boundary condition *C*_0_^emergent^, the functional weighting construct *η*(*t*), and the candidate derived exposure metric *E*_functional_, and translates the framework into four falsifiable predictions, while **Table 1** specifies the measurements required to evaluate them.

A practical implication for the field is therefore methodological. Early-phase *in vivo* CAR-T trials should report not only conventional cellular kinetic readouts but also the generation-phase variables that determine *C*_0_^emergent^. Whether the production-rate framework holds, requires revision, or is replaced by a successor framework will be settled by data — as it should be.

## Supplementary material

Supplementary Table 1 Evidence base, highest evidence level, principal uncertainty, and principal safety concern for each in vivo CAR-T platform class.
