# Supplementary material for: In vivo CAR-T cell generation: a production-rate framework linking vector and cellular pharmacology
Source: Front Immunol. 2026 Sep 18;17:1939127. doi: 10.3389/fimmu.2026.1939127 (PMC13631744; doi:10.3389/fimmu.2026.1939127)
Supplement: Supplementary file 1 [file Table1.docx]

Supplementary Table S1. Evidence maturity, principal uncertainties, and safety considerations across *in vivo* CAR-T generation platforms.

| Platform category | Evidence base to date | Highest evidence level | Principal uncertainty | Principal safety concern |
| --- | --- | --- | --- | --- |
| I. mRNA–LNP | Murine and NHP evidence supports targeted mRNA–LNP-mediated CAR-T generation.^9,10^ A 2026 first-in-human conference abstract reported that seven patients with relapsed or refractory B-cell lymphoma had been treated with a CD8-targeted LNP encoding a CD19 CAR as of the reported data cutoff, using an initial intravenous dose followed by three boost doses at 3-day intervals; three patients were evaluable for response. A full-length peer-reviewed clinical report was not available at the evidence-cutoff date.^84^ | First-in-human  (conference abstract) | Durability of clinical activity and the safety, pharmacokinetics, and immunogenicity of repeat dosing in larger cohorts and with longer follow-up | No cytokine release syndrome of grade 2 or higher was reported in the first-in-human cohort.^84^ LNP reactogenicity and innate immune activation; formulation-dependent anti-PEG immunity and altered pharmacokinetics or tolerability with repeat systemic dosing.^34,72^ |
| II. circRNA-enabled | CAR-encoding circRNA has been evaluated in primary human T cells *ex vivo*, with antitumor activity of the resulting cells demonstrated after adoptive transfer in murine models.^62^ Direct systemic *in vivo* CAR-T generation using CAR circRNA delivered by CD7-targeted LNPs has been demonstrated in a murine autoimmune-disease model.^85^ | Murine *in vivo* proof-of-concept; no NHP or clinical evidence | Reproducibility across targeting ligands, CAR constructs, disease settings, and species; generation kinetics, redosability, and durability in large-animal and human studies | No clinical safety evidence; innate sensing of circRNA, off-target expression, delivery-system reactogenicity, and repeat-dose immunogenicity remain to be characterized.^62,85^ |
| III. Non-viral DNA + transposon | Murine *in vivo* CAR-T generation using targeted LNP delivery of CAR-encoding mcDNA and SB100x transposase mRNA; *ex vivo* preclinical evidence for Sleeping Beauty-engineered CAR-cell products.^64,65^ | Murine *in vivo* | *In vivo* integration efficiency and per-cell copy number | Potential insertional genotoxicity associated with semi-random integration, variable per-cell copy number, and uncertainty regarding the magnitude and duration of transposase exposure.^64,65^ |
| IV. Targeted LVV | Two successive peer-reviewed reports from a single development program (a four-patient case series and a subsequent completed five-patient phase 1 study), together with a preliminary conference report from an independent program and NHP evidence.^11–13,37^ | First-in-human peer-reviewed early clinical evidence | Interpatient variability in emergent cellular output at fixed vector dose; durability beyond short follow-up; the completed phase 1 study was stopped early after five patients, limiting cohort size and follow-up | In the five-patient ESO-T01 phase 1 study, all five patients experienced grade ≥3 adverse events and CRS occurred in four patients. Additional platform-level concerns include semi-random genomic integration and immune responses that may limit repeat dosing.^12,36,37^ |
| V. AAV monotherapy | Direct AAV-CAR monotherapy has been demonstrated in a humanized mouse model.^66^ Separate murine evidence shows that the evolved AAV variant Ark313 can deliver transgenes and gene-editing components to T cells *in vivo*, but this does not constitute direct evidence for AAV-CAR monotherapy.^67^ | Murine *in vivo*; direct CAR evidence limited to a humanized mouse model | Whether AAV monotherapy can achieve therapeutically meaningful CAR-T generation and persistence in proliferating T cells | General AAV-platform concerns include pre-existing neutralizing antibodies and capsid-specific T-cell responses; their relevance to AAV-mediated *in vivo* CAR-T generation has not been clinically defined.^56–58^ |
| VI. Site-specific integration | *In vivo* site-specific engineering of human T cells has been demonstrated in humanized mouse models, including solid-tumor models; no human clinical evidence is available.^14^ Separate *ex vivo* and *in vivo* murine T-cell-targeting evidence supports the evolved AAV donor component.^48,67^ | Murine *in vivo* evidence, including humanized mouse models | Efficiency, specificity, and durability of editing human T cells in patients; dual-vector biodistribution, temporal overlap, and productive co-uptake | Off-target editing, unintended donor integration or other genomic alterations, and the irreversibility of successfully edited cells despite transient delivery of the editing components.^14^ |

This table complements Table 1: Table 1 summarizes platform-level design attributes, whereas entries here indicate the maturity of the supporting evidence. Evidence sources were updated through 21 August 2026. Conference abstracts are classified separately from full-length peer-reviewed clinical reports. Evidence level should not be interpreted as equivalent to clinical readiness, therapeutic validation, or evidence that all platform-level attributes have been demonstrated at that level. Evidence derived from *ex vivo* engineering, reporter expression, or individual delivery components is explicitly distinguished from direct *in vivo* CAR-T generation. Reference numbers correspond to the reference list in the main manuscript.

Abbreviations: AAV, adeno-associated virus; CAR, chimeric antigen receptor; circRNA, circular RNA; CRS, cytokine release syndrome; LNP, lipid nanoparticle; LVV, lentiviral vector; mcDNA, minicircle DNA; NHP, non-human primate; PEG, polyethylene glycol; SB100x, hyperactive Sleeping Beauty transposase.
